# Preferred and avoided codon pairs in three domains of life

**DOI:** 10.1186/1471-2164-9-463

**Published:** 2008-10-08

**Authors:** Age Tats, Tanel Tenson, Maido Remm

**Affiliations:** 1 Department of Bioinformatics, Institute of Molecular and Cell Biology, University of Tartu, Riia str. 23, Tartu 51010, Estonia; 2 Institute of Technology, University of Tartu, Nooruse str. 1, Tartu 50411, Estonia; 3 Estonian Biocentre, Riia str. 23, Tartu 51010, Estonia

## Abstract

**Background:**

Alternative synonymous codons are not used with equal frequencies. In addition, the contexts of codons – neighboring nucleotides and neighboring codons – can have certain patterns. The codon context can influence both translational accuracy and elongation rates. However, it is not known how strong or conserved the codon context preferences in different organisms are. We analyzed 138 organisms (bacteria, archaea and eukaryotes) to find conserved patterns of codon pairs.

**Results:**

After removing the effects of single codon usage and dipeptide biases we discovered a set of neighboring codons for which avoidances or preferences were conserved in all three domains of life. Such biased codon pairs could be divided into subtypes on the basis of the nucleotide patterns that influence the bias. The most frequently avoided type of codon pair was nnUAnn. We discovered that 95.7% of avoided nnUAnn type patterns contain out-frame UAA or UAG triplets on the sense and/or antisense strand. On average, nnUAnn codon pairs are more frequently avoided in ORFeomes than in genomes. Thus we assume that translational selection plays a major role in the avoidance of these codon pairs. Among the preferred codon pairs, nnGCnn was the major type.

**Conclusion:**

Translational selection shapes codon pair usage in protein coding sequences by rules that are common to all three domains of life. The most frequently avoided codon pairs contain the patterns nnUAnn, nnGGnn, nnGnnC, nnCGCn, GUCCnn, CUCCnn, nnCnnA or UUCGnn. The most frequently preferred codon pairs contain the patterns nnGCnn, nnCAnn or nnUnCn.

## Background

The frequencies of synonymous codons in protein coding sequences are biased and different organisms tend to use different sets of synonymous codons. In addition, other codons are juxtaposed non-randomly with each codon. These preferences are typically referred to as codon context biases. It is suggested that codon context biases are associated with translational efficiency, since codon context influences translational elongation rates [1]. Moreover, experimental results support the observation that codon context is more strongly related to translational efficiency than single codon usage [1]. A second important parameter that is influenced by codon context is translational accuracy. Codon context can influence both mis-sense and nonsense suppression [2-7]. In addition, codons in combination with surrounding nucleotides can form mononucleotide repeats, which may cause transcriptional [8,9] or translational [10] slippage. Frameshift errors on 'hungry' codons in specific nucleotide contexts also increase under starvation conditions [11]. Several programmed frameshifting sites have been described in the coding regions of mRNAs from different organisms (*e.g. *[12,13]). Such sites are used for regulating gene expression through recoding. Nevertheless, frameshifting errors are rare events in most sequences, occurring with a frequency less than once every 10,000 codons [14]. This means that sequences that are prone to frameshifting are successfully avoided in coding sequences. For example, it has been shown that certain heptanucleotides that are prone to frameshifts are under-represented in the coding sequences of *Saccharomyces cerevisiae *[15] and *Escherichia coli *[10].

There have been many studies analyzing codon pair biases in a limited number of species [16-21]. The main selective effects on codon context are found in the nucleotides following the codon in the 3' direction [16,18,19,22]. It has been found that the specific preferred or avoided nucleotide patterns differ among species [16,22].

The only large-scale comparative analysis to date suggested that the codon context in eukaryotes is biased because target sequences for DNA methylation and trinucleotide repeats are present at high frequencies, while in bacteria and archaea the codon context is influenced mainly by the translational machinery [22].

Since the structure and function of the ribosomal decoding centre are highly conserved in evolution, we could expect that avoidance of and preference for certain sequence contexts would also be conserved in the protein coding sequences of different organisms. Previous studies suggest that the effects of codon context are influenced by the physical interactions between tRNA isoacceptors in the ribosomal P- and A-sites [1,16,23]. It has been shown that in five gamma proteobacteria, *Bacillus subtilis *and two yeasts, the A-site codons decoded by the same tRNA have similar patterns of P-site codon pairing preference [16]. In addition, it has been confirmed that E-site occupation is essential for preventing frameshift [24-26]. It was shown recently that the species specific combinations of three consecutive codons are highly biased among fungal species and even reaching to the complete vanishing of certain combinations [27]. This study is a comparative analysis of the usage of neighboring and more distant codon pairs in 138 randomly selected organisms belonging to different domains of life. We show that certain codon context biases are conserved in the protein coding sequences of different species. Most of them are probably influenced by translational rather than DNA-related mechanisms.

## Results

### Definition of conserved biased codon pair

To study the common rules of codon context bias we looked for codon pairs that are significantly preferred or avoided in the three domains of life. These conserved cases of biased codon pairs are most probably caused by conserved molecular mechanisms and may perhaps shed light on the mechanisms shaping the genes and genomes. In this study a preferred or avoided codon pair was designated a "conserved biased codon pair" if it was statistically significantly avoided or preferred in more than 50% of the organisms studied. This criterion is likely the reason why we found many more conserved codon pairs as opposed to other findings [22], where the universal rules were searched only among the first ten most conserved codon pairs in each separate domain.

The significance of the bias of each codon pair in each genome was calculated by comparing the observed and expected occurrences of that pair in the open reading frames (ORFs) of a given genome (see Methods). It is important to emphasize that the expected frequency of a codon pair represents the random co-occurrences of two codons, not the expected frequency of the corresponding hexanucleotide. This means that the significantly over-represented co-occurrence of two codons does not necessarily imply that the corresponding hexanucleotide sequences occur with high frequency.

It is known that proteins contain certain dipeptides at increased and reduced frequencies [17]. To ensure that the effects observed at the codon pair level were not caused by avoidances or preferences of dipeptides, the expected codon pairs values were normalized to the dipeptide frequencies (see Methods). This aspect was not considered in previous studies [21,22].

On the basis of that criterion, we found 288 neighboring codon pairs (1–2 codon pairs) that were preferred or avoided in most of the organisms studied [Additional files 1, 2]. We also tested the conservation of more distant (1–3, 1–4, 1–5) codon pairs. However, for codons 1–3 we found only one codon pair with significant bias – GGUnnnGGU – which was over-represented in 61% of the organisms studied. No conserved biases were found for more distant codon pairs. Thus, all the following analyses are based on neighboring (1–2) codon pairs.

### Method for comparing ORFeomes and genomes

The most straightforward method for testing the translational effects of under- and over-representation of a codon pair would be to compare its avoidances and preferences in the correct reading frame and in two other reading frames. In such a comparison, however, one cannot effectively remove the influence of single codon preferences or amino acid preferences on the avoidance/preference of codon pairs in other frames. Thus, we consider the comparison of effects in +1 and +2 reading frames biased and incorrect, so we compare the effects in the ORFeome and genome instead.

Therefore, to test whether codon pairs are biased because of translational effects or because of mechanisms operating at the DNA level, we calculated the ratio of observed and expected co-occurrences of two trinucleotides at the genome level. If the main selective force for codon pair usage were related to translational effects at the ribosome and not to biases in mechanisms operating at DNA level, the codon pair bias would be stronger in ORFeomes than in genomes. Some conserved under-represented codon pairs contained the stop-codon in +1 or +2 frame. Corresponding trimers cannot occur in one of the frames in genomic regions where they overlap ORFs. This reduced frequency of occurrence was taken into account when the ratio of observed to expected co-occurrences of trimers in genomes was determined.

### Under-represented codon pairs can be divided into 9 major types

We identified 207 codon pairs that were avoided in the ORFs of more than half the organisms studied (Table 1, [additional file 1]). To elucidate the molecular mechanisms causing these avoidances, we tried to find recurring sub-patterns in the conserved biased codon pairs and to classify them according to those sub-patterns. Unfortunately, the magnitude of the effects (observed/expected ratio) of pentamers, tetramers, trimers and dimers cannot be compared directly with that of codon pairs because the number, and therefore the variation in magnitude of effect, differs markedly among sub-patterns of different lengths [Additional file 3]. To overcome this problem we calculated the standard deviation for each sub-pattern family and used it to normalize the magnitudes of effects. This led to a score for each codon pair and sub-pattern that described the divergence of the observed/expected ratio from the mean in units of standard deviation. This score is traditionally called the z-score. Z-scores make sub-patterns of different lengths more comparable to each other and can be used to identify the sub-pattern level on which the effect is strongest.

**Table 1:** The top 10 most conserved avoided codon pairs in the organisms studied

12 ↓ codon pairs	%	log_2_(obs/exp)		A – B	type
				
		ORFeome (A)	genome (B)		
UUCGCA	86	-0.81	-0.86	0.05	6_A_
GGGGGU	83	-1.12	-0.43	-0.69	8_A_
UUCGAA	82	-0.76	-0.75	-0.01	6_A_
**CUUAUG**	79	-0.92	-0.63	-0.29	1_A_
**GCUAUG**	76	-0.76	-0.28	-0.48	1_A_
**ACUAUG**	73	-0.71	-0.21	-0.50	1_A_
**GUUAGC**	73	-0.92	-0.52	-0.40	1_A_
**CUUAGU**	73	-0.94	-0.83	-0.11	1_A_
UUCGCG	72	-0.84	-0.56	-0.28	3_A_
**GUUAUG**	72	-0.71	-0.30	-0.41	1_A_

Codon pairs containing out-frame UAA or UAG triplets on the sense and/or antisense strand are marked in bold. The observed/expected ratios in logarithmic scale for each codon pair in the ORFeome and genome are shown. % – the percentage of organisms in which the codon pair is significantly avoided. A – B – difference between log_2 _(obs/exp) ratios in the ORFeome and the genome. A – B < 0 represents a stronger effect on the ORFeome level. For the avoided pattern types see Table 2. For the full lists of codon pairs avoided in at least 50% of the organisms studied see [additional file 1].

The avoided codon pairs could be divided into 9 major groups (Table 2, [additional file 4]). The most abundant types of under-represented pairs were nnUAnn (Type 1_A_). Among the avoided nnUAnn codon pairs, 75.7% were more strongly avoided on average in the ORFeome than in the genome (Table 2). This suggests that selection for nnUAnn avoidance occurs mainly at the translational level. This universal effect is clearly visible in the human genome, where the nnUAnn type codon pairs were also less frequent than expected (77.1% of nnUAnn type pairs were more strongly avoided in the human ORFeome than in the genome).

**Table 2:** Types of patterns among conserved avoided codon pairs

avoided pattern		% among avoided pairs	the effect is stronger in ORFeome (%)	the effect is stronger in whole genome (%)
type 1_A_	nnUAnn	33.8	75.7	24.3
type 2_A_	nnGnnC	13.6	100.0	0.0
type 3_A_	nnCGCn	8.7	77.8	22.2
type 4_A_	(G/C)UCCnn	6.8	100.0	0.0
type 5_A_	nnCnnA	6.3	69.2	30.8
type 6_A_	UUCGnn	3.9	75.0	25.0
type 7_A_	nnGGnn	3.9	100.0	0.0
type 8_A_	mononucleotide repeats	3.9	100.0	0.0
type 9_A_	nCAUAn	1.9	100.0	0.0

The percentage of codon pairs of the corresponding pattern among all conserved avoided codon pairs is shown. For specific codon pairs of each type see [additional file 4].

Many of the nnUAnn type patterns contained codon pairs with stop codons in -1 frame on the sense strand (67%, 47/70). For such pairs, a -1 frameshift event would create premature translational termination. Thus, we assume that avoidance of nnUAnn codon pairs is partly related to out-frame stop codons. On the other hand, avoidance of the UA dinucleotide between two codons also has a role here, because out-frame UGA stop-codons were not observed in any of the conserved biased dicodon pairs.

Interestingly, 83% (58/70) of the nnUAnn type patterns contained out-frame UAA and UAG triplets on the antisense strand. However, there are no known mechanisms that could explain the avoidance of UAA and UAG triplets in the middle of nnUAnn type hexamers on the antisense strand.

Including the antisense strand, almost all (67/70, 95.7%) of the nnUAnn type patterns contained UAA or UAG in -1 frame, although nnUAnn could code for other hexamers containing UAU and UAC in 25% of cases. Only three of the type 1_A _codon pairs did not contain out-frame UAA or UAG. All three began with GGUA and did not show strong avoidance on the ORFeome level. Those three pairs may not be related to the same kind of avoidances as all other type 1_A _codon pairs [Additional file 4].

The second most abundant type of conserved avoided codon pair was nnGnnC (type 2_A_), which was more strongly avoided in ORFeomes than in genomes. The third type, type 3_A _contained the pattern nnCGCn. Avoidance of mononucleotide repeats such as GGGGGn, GGGGnn, nCCCCn and UUUUUU was also conserved in most organisms (Type 8_A_). UUUUUU was clearly avoided in ORFeomes but was significantly preferred at the genome level (Table 2).

The most conserved avoided codon pair was UUCGCA (type 6_A_, UUCGnn), which was under-represented in 86% of the organisms studied. It is interesting to note that this codon pair contains several clearly avoided sub-patterns (UUCGnn, nnCGCn, nnCnnA). The observed/expected ratios of this pair in the ORFeome and genome indicated that UUCGCA was similarly under-represented on both the ORFeome and genome levels (Table 1).

Interestingly, among the different avoided sub-patterns causing the conserved avoidance of codon pairs, the last nucleotide of the P-site codon in a pair was always fixed (the only exception was the pattern UnnnnU for codon pair UUUUUU).

### Over-represented codon pairs can be divided into 4 types

We found 81 codon pairs that were over-represented in more than half the organisms studied (Table 3, [additional file 2]). Four major preferred types can be described: nnGCnn, nnCAnn, nnUUnn and nnUnCn (Table 4). The most abundant type of conserved over-represented codon pair was nnGCnn (Type 1_P_). All the major types were more strongly over-represented in ORFeomes than in genomes, again indicating that the common preference of codon pairs that we detected is mainly influenced by translational mechanisms. The most conserved preferred codon pair, GGGCUU, was over-represented in 76% of the organisms studied and also belonged to Type 1_P_.

**Table 3:** The top 10 most conserved preferred codon pairs in the organisms studied

12 ↑ codon pairs	%	log_2_(obs/exp)		A – B	type
				
		ORFeome (A)	genome (B)		
GGGCUU	76	0.99	0.44	0.55	1_P_
GAGCAG	70	0.51	0.64	-0.13	1_P_
GGGCAU	69	0.83	0.39	0.44	1_P_
**UUUGAA**	66	0.31	0.12	0.19	
GACAGC	64	0.70	0.21	0.49	2_P_
UUUGCC	64	0.51	0.07	0.44	4_P_
GGAACA	64	0.82	0.52	0.30	
UACAAC	64	0.58	0.36	0.22	2_P_
**AUCAUC**	64	0.42	0.53	-0.11	2_P_
CUUUCU	61	0.91	0.29	0.61	3_P_

Codon pair containing out-frame UAA or UAG triplets on the sense and/or antisense strand are marked in bold. The observed/expected ratios in logarithmic scale for each codon pair in the ORFeome and genome are shown. % – the percentage of organisms in which the codon pair is significantly preferred. A – B – difference between log_2 _(obs/exp) ratios in the ORFeome and the genome. A – B > 0 represents a stronger effect on the ORFeome level. For the preferred pattern types see Table 4. For the full lists of codon pairs preferred in at least 50% of the organisms studied see [additional file 2].

**Table 4:** Types of patterns among conserved preferred codon pairs

preferred pattern		% among avoided pairs	the effect is stronger in ORFeome (%)	the effect is stronger in whole genome (%)
type 1_P_	nnGCnn	21.0	70.6	29.4
type 2_P_	nnCAnn	13.6	63.6	36.4
type 3_P_	nnUUnn	11.1	100.0	0.0
type 4_P_	nnUnCn	9.9	75.0	25.0

The percentage of codon pairs of the corresponding pattern among all conserved preferred codon pairs is shown. For specific codon pairs of each type see [additional file 5].

As in the conserved avoided codon pairs, all the different preferred sub-patterns that caused codon pair preferences contained a fixed last nucleotide of the P-site codon in the pair.

### Phylogenetic distribution of the conserved codon context patterns

**Figure 1:**
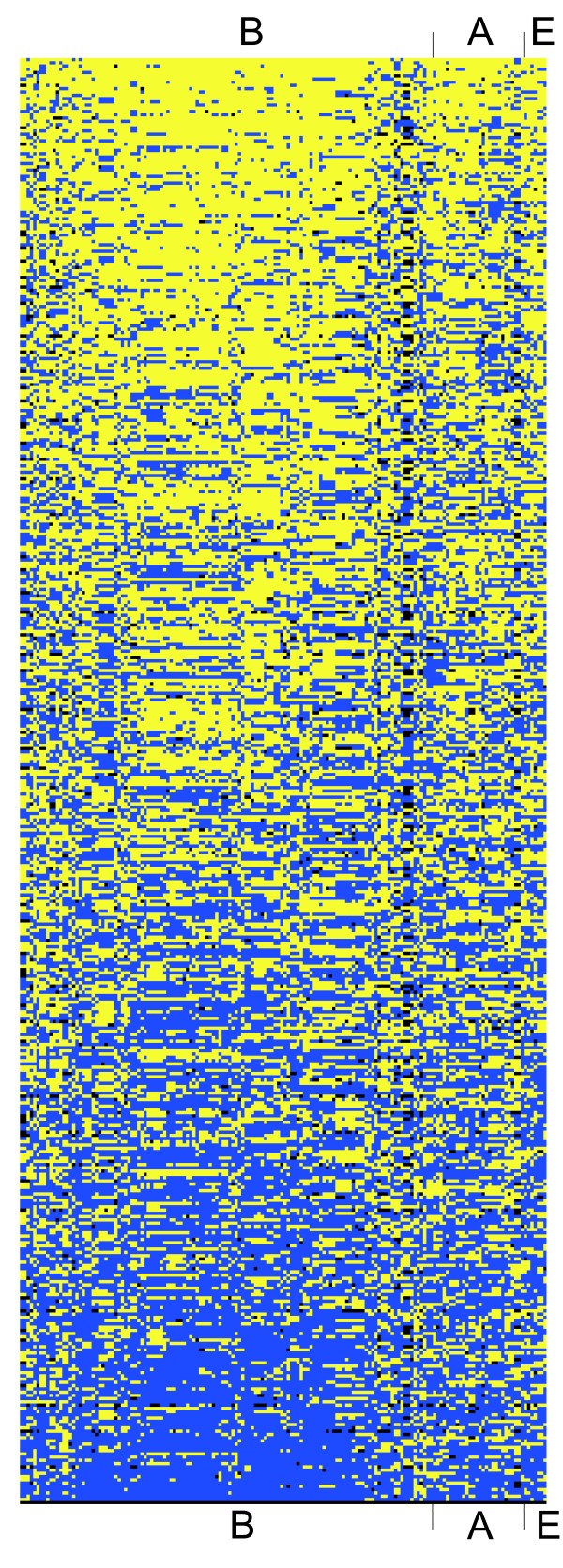
**The most avoided and the most preferred codon pairs are uniformly distributed in all three domains of life**. The map is clustered on the basis of the conservation of avoidance and preference of different codon pairs. The avoided codon pairs are marked in yellow (obs/exp < 0). The preferred codon pairs are marked in blue (obs/exp > 0). Codon pairs without bias are black (obs/exp = 0). No additional criteria were applied to the figure. Codon pairs are ranked downwards according to the decreasing conservation of avoided codon pairs in the organisms studied. B – bacteria, A – archaea, E – eukaryotes.

**Figure 2:**
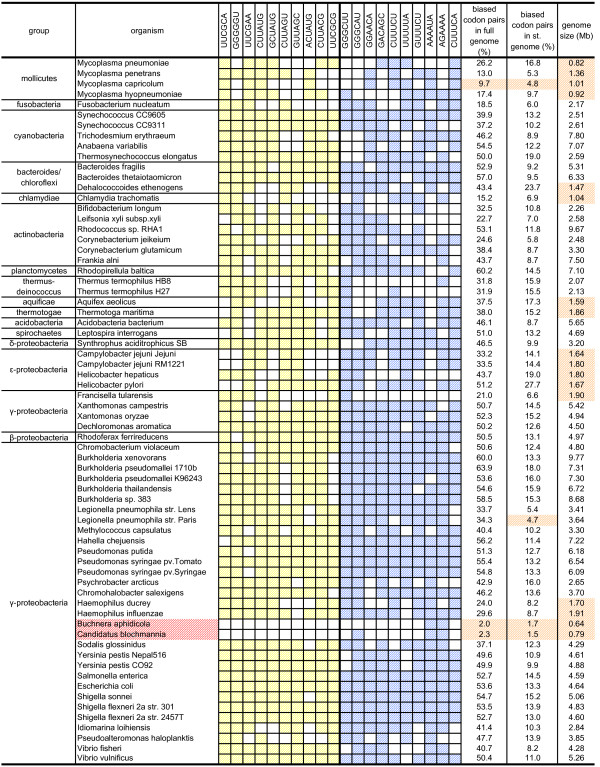
**The distribution of the top ten significantly under-represented and the top ten significantly over-represented codon pairs in the organisms studied**. The colored cells mark significant bias of the pattern in the corresponding organisms (observed/expected ≤ 0.90 (yellow) or observed/expected ≥ 1.10 (blue), p-value ≤ 0.01). Names of organisms with fewer than five biased codon pairs out of 20 are colored red. The percentages of biased codon pairs in full genomes and in standardized genomes and genome sizes are also shown. Yellow shaded cells – less than 10% of biased codon pairs in full genomes; less than 5% of biased codon pairs in standardized genomes; genomes smaller than 2 Mb. st. genome – standardized genome.

**Figure 3:**
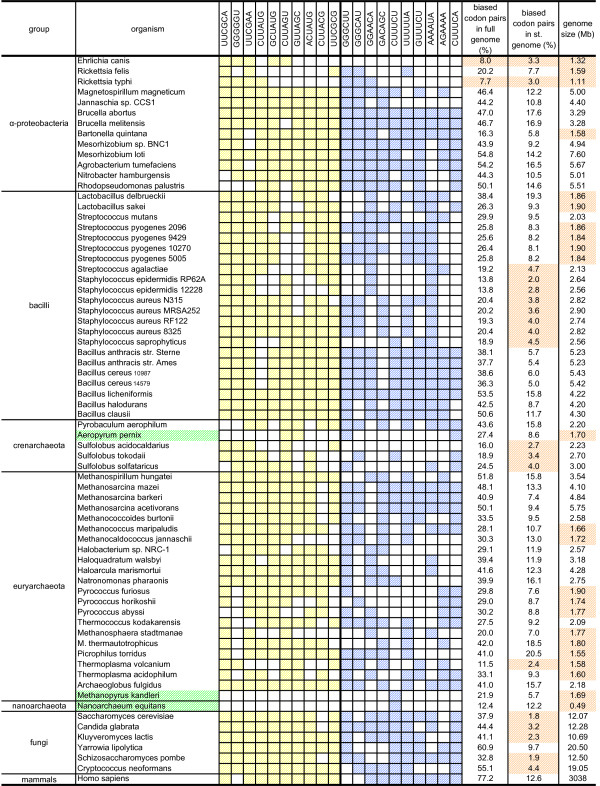
**The distribution of the top ten significantly under-represented and the top ten significantly over-represented codon pairs in the organisms studied (continuation of Figure 2)**. The colored cells mark significant bias of the pattern in the corresponding organisms (observed/expected ≤ 0.90 (yellow) or observed/expected ≥ 1.10 (blue), p-value ≤ 0.01). Names of organisms having fewer than five biased codon pairs out of 20 are colored green. The percentages of biased codon pairs in full genomes and in standardized genomes and genome sizes are also shown. Yellow shaded cells – less than 10% of biased codon pairs in full genomes; less than 5% of biased codon pairs in standardized genomes; genomes smaller than 2 Mb. st. genome – standardized genome.

How are the most preferred and avoided codon pairs distributed among different phylogenetic classes of organisms? To estimate the distribution of biased codon pairs between phylogenetic groups we built a cluster map of all codon pairs in the organisms studied (Figure 1). It can be seen that the most avoided and the most preferred codon pairs are uniformly distributed across all three domains of life. To investigate this more closely, we plotted the ten most conserved and under-represented and the ten most conserved and over-represented codon pairs against a phylogenetically organized list of all the organisms studied (Figures 2 and 3). Although phylogenetically very close species tend to have similar codon pair usage, no major phylogenetic group-specific distribution was observed. This indicates that the under- and over-represented codon pairs are indeed uniformly distributed.

### Five genomes had atypical sets of biased codon pairs

Interestingly, five organisms showed significant bias in fewer than five of the top 20 codon pairs (Figures 2 and 3). This raised the question: do those five genomes have different sets of most-biased codon pairs, do they lack strong codon pair biases as such, or do we just lack the statistical power to detect biased codon pairs in them?

**Figure 4:**
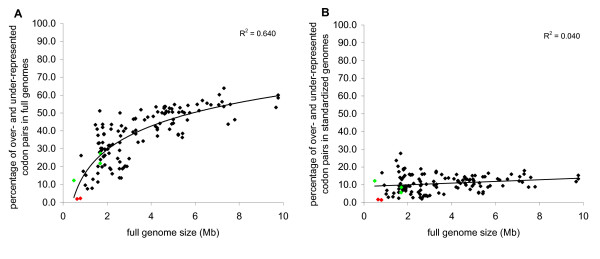
**The effect of genome size on the fraction of biased codon pairs**. **A **– the percentage of biased codon pairs in full bacterial genomes. **B **– the percentage of biased codon pairs in standardized bacterial genomes. Green diamonds – *Aeropyrum pernix*, *Methanopyrus kandleri *and *Nanoarchaeum equitans*. Red diamonds – *Buchnera aphidicola *and *Candidatus blochmannia pensilvanicus*.

To distinguish among these possibilities we calculated the percentage of biased codon pairs in all the genomes under study. It appeared that those five organisms can be split into two groups. *Aeropyrum pernix*, *Methanopyrus kandleri *and *Nanoarchaeum equitans *had a considerable number of biased codon pairs (Figure 3), indicating that these organisms use a different set of biased codon pairs from the conserved set. However, *Buchnera aphidicola *and *Candidatus blochmannia pensilvanicus *had bias in only 2.0% and 2.3% of codon pairs, respectively, as compared to the average 37.7% (Figure 2). This suggests that those genomes either essentially lack codon pair bias or we lack the statistical power to detect their biased codon pairs. The genomes of *B. aphidicola *and *C. blochmannia *were also among the smallest in our study. It is possible that smaller genomes do not have enough codon pairs to reach statistical significance under the criteria we applied. Indeed, larger genomes appeared to contain larger fractions of biased codon pairs than smaller genomes (Figure 4A). Could the small number of biased codon pairs in *B. aphidicola *and *C. blochmannia *simply be the result of a low detection limit in small genomes?

To answer this question, we created a dataset containing 150,000 randomly sampled codon pairs from all the genomes studied. This should correspond to the genome size 0.45 Mb, which is close to the smallest genome in our set. Using this standardized genome dataset we calculated how many codon pairs would still remain significantly biased (Figures 2 and 3). The results show that genome size indeed has a statistical effect on the number of biased codon pairs detected. The fraction of biased codon pairs leveled off after genome reduction (Figure 4B). However, the same figure also demonstrates that genome size was not the reason why *B. aphidicola *and *C. blochmannia *have low numbers of biased codon pairs. Even in the standardized sample, most other genomes showed bias in 5–15% codon pairs, whereas *B. aphidicola *and *C. blochmannia *had only 1.5% and 1.7% biased codons respectively. Therefore, these two genomes seem effectively to lack biased codon pairs.

We conclude that codon pair usage bias can be distributed in many different ways. Although most organisms have a similar set of universally conserved biased codon pairs, some organisms use slightly different sets (*e.g. N. equitans*) and some have a very small number of biased codon pairs (*e.g. B. aphidicola, C. blochmannia*).

### Evolutionary conservation of codon context

Our findings suggest that certain codon contexts are strongly conserved over all domains of life. It has been proposed that codon context is even more important than single codon usage for translational efficiency [1].

**Figure 5:**
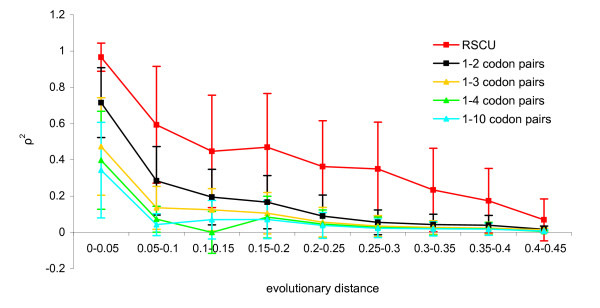
**Correlation of codon usage and codon context usage in bacteria**. RSCU – relative synonymous codon usage; 1–2 – neighboring codon pairs; 1–3 – codons separated by one intervening codon; 1–4 – codons separated by two intervening codons; 1–10 – codons separated by eight intervening codons. Evolutionary distances between bacteria were retrieved as a 16SRNA distance matrix from the Ribosomal Database Project [28]. *ρ*^2 ^– Spearman's correlation coefficient.

To analyze whether single codon preference or codon pair preference is more conserved on the evolutionary scale, we compared different bacteria according to RSCU (relative synonymous codon usage) and RDCU (relative dicodon usage). Similarity was measured by calculating the correlation (Spearman's *ρ*) of RSCU and RDCU values between each pair of bacteria. All pairs of bacteria analyzed were divided into nine groups according to the evolutionary distance separating each pair. Pairwise evolutionary distances were retrieved as a 16SRNA distance matrix from the Ribosomal Database Project [28]. The average correlation coefficients of RSCU and RDCU were calculated for each group. We observed that the correlation of RSCU values was generally higher than the correlation of RDCU values (Figure 5). As expected, the highest correlation of RSCU occurred in the phylogenetically closest bacteria. The greatest similarity in RDCU among the species analyzed occurred when the calculation was based on 1–2 codon pairs. Extending the distance between two codons (1–3, 1–4, 1–10) decreased the RDCU correlation.

Codon pair usage analysis showed that the most considerably biased conserved codon pairs are biased irrespective of phylogenetic class (Figures 1, 2, 3). To determine whether this is also true when the usage of all possible codon pairs is considered and compared with RSCU in different phylogenetic classes, we used a tree-based method. The correlation of RSCU or RDCU usage between two organisms can be used as a measure of the distance between them: the higher the correlation, the shorter the distance. For example, the distance between two organisms with identical codon usage would be 0 (1-*ρ*^2 ^with *ρ*^2 ^= 1). These distances can be represented as trees and compared to ribosomal RNA sequence-based phylogenetic trees.

**Figure 6:**
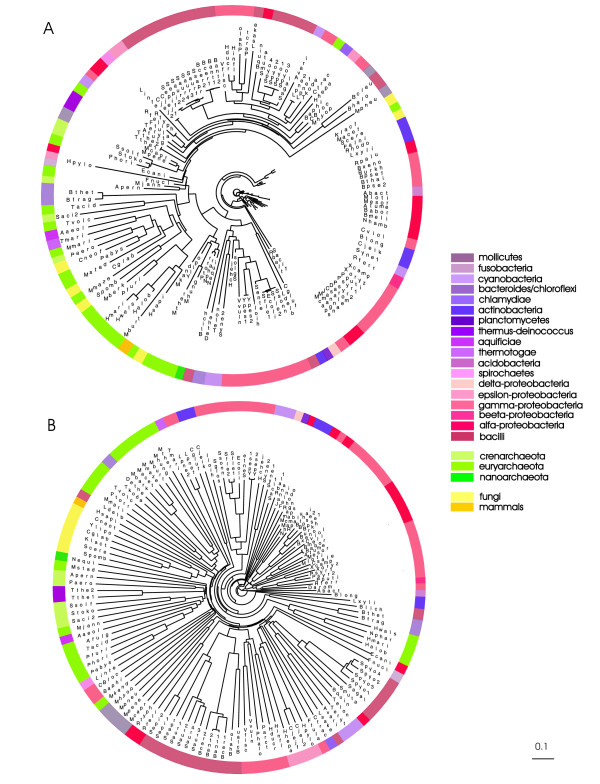
**The evolutionary conservation of RSCU (A) and RDCU (B) in the genomes studied**. The branch lengths characterize the correlation of RSCU or RDCU usage between two organisms – the higher the correlation, the shorter the branch. In general, the branch lengths of the RSCU tree are shorter than those of the RDCU tree, showing stronger similarity of codon usage than codon pair usage between different organisms. Bacilli, gamma-proteobacteria and alpha-proteobacteria form very similar clusters on both trees. Eukaryotes, which are spread around the RSCU tree, are all clustered together in the RDCU tree. For the full names of organisms see [additional file 6].

Comparing the RSCU and RDCU trees, we observed that the branch lengths were shorter in the RSCU tree (Figure 6A) than in the RDCU (codons 1–2) tree (Figure 6B), showing that codon usage gives stronger similarity between different organisms than codon pair usage. However, only a few clearly-separated phylogenetic classes occurred in both trees. Some were similarly clustered in both trees, *e.g. *bacilli, gamma-proteobacteria and alpha-proteobacteria. This indicates that in addition to similar codon usage, these organisms use similar codon contexts. In contrast, eukaryotes showed different patterns, being spread around the codon usage tree (Figure 6A), but clustered together in the codon context tree (Figure 6B). This suggests that between different eukaryotes (which in our dataset were mostly represented by fungi) the similarity in codon context is greater than the similarity in codon usage. Still, it has to be noted that the sample we used for eukaryotes is not representative since it is small and contains only one mammal.

## Discussion

The current study is an extensive investigation of sequence context patterns that are independent of single codon usage and dipeptide usage. We found that some combinations of neighboring codons are similarly avoided or preferred in many different organisms – bacteria, archaea and eukaryotes. The conserved avoidances and preferences of codon pairs observed are not the result of dipeptide biases since the effect of dipeptides was removed. Much of the dataset could be divided into subtypes on the basis of nucleotide patterns influencing the bias of codon pairs. Conserved patterns result mainly from translational effects not from DNA-related mechanisms since the biases are stronger in ORFeomes than in genomes.

It was claimed previously that codon pair preference is primarily determined by a tetranucleotide combination including the last nucleotide of the P-site codon and all three nucleotides of the A-site codon [16]. However, our results showed that different patterns ranging from dinucleotides to hexanucleotides could explain conserved biased codon pair usage. Still, with one exception (codon pair UUUUUU and pattern UnnnnU), all sub-patterns contained a fixed nucleotide in the last position of the P-site codon in the codon pair. As the ribosome does not contact the bases of the codon in the P-site [29], the reason for this potential P-site effect is not clear.

Previously, the only universal context selection rule found to cover all three domains of life was the avoidance of most codon pairs of the nnUAnn type, which was suggested to result from rejection of TA dinucleotides in DNA sequences [22]. Among 9 groups of under-represented codon pairs found in our study the largest group was also influenced by the avoidance of UA dinucleotides. However, although TA dinucleotides could be avoided at the genome level, this would not exclude the possibility that avoidance of UA dinucleotides is also important for ORFs and effective translation. Our methods allowed us to compare the observed/expected ratios of codon pairs more specifically between ORFeomes and genomes. The results showed that in 75.7% of cases the avoidance effect for nnUAnn codon pairs was stronger in ORFeomes than in genomes, suggesting the influence of translational mechanisms (Table 2).

Many of the avoided nnUAnn patterns contained out-frame stop codons, UAA or UAG, on the sense strand. This indicates that out-frame stop codons influence the avoidance of nnUAnn codon pairs. The reason for avoiding the codon pairs containing out-frame stops could be to minimize premature translational termination through recognition of those stops by a translation termination factor. The observation that only the stop codons UAA and UAG were avoided suggests that this kind of misreading might be caused by termination factor 1, the protein responsible for decoding them. Although the frequencies of erroneous termination events have been studied [30], we have no information concerning the possible recognition of termination codons through a frameshifting event. Interestingly, most nnUAnn codon pairs also contained out-frame UAA and UAG triplets on the antisense strand. There are no known mechanisms that could explain such avoidances. However, our results suggest that nnUAnn type avoidances are related to translational mechanisms because they are stronger in ORFeomes than in genomes.

Mononucleotide repeats, especially poly(A) and poly(U) tracts, are also known to cause transcriptional and translational frameshifting [8-10]. Therefore, such contexts should be selected against in protein coding sequences. There were eight mononucleotide repeats among the avoided codon pairs. The repeated nucleotide was G (GGGGGG, GGGGGU, GGGGGC and GGGGCC), C (ACCCCG, ACCCCA and GCCCCG) or U (UUUUUU) [Additional file 4]. All those codon pairs were more strongly avoided in ORFeomes than in genomes. This suggests that they were avoided to reduce the frequency of frameshifting events in polynucleotide sequences.

We also found several conserved preferred codon pairs. The number of conserved preferred codon pairs was smaller than the number of avoided codon pairs [Additional files 1, 2]. This suggests that the selection for more effective and more accurate translation acts primarily through avoidance of the most disadvantageous codon pairs and not through over-representation of the most suitable contexts. The most prevalent type of conserved preferred codon pair was nnGCnn [Additional file 5].

The top 10 avoided and preferred codon pairs were not specific to any larger phylogenetic group, suggesting that usage of those codon pairs is universally conserved (Figures 2 and 3). However, in some organisms with small genomes, only a few of those 20 codon pairs were significantly biased. This is not caused by statistical limitations on finding biased codon pairs in smaller genomes, but rather by the absence of codon-pair bias in those organisms. We also observed that some genomes use sets of most avoided and most preferred codon pairs different from the conserved sets identified in this study.

It has been proposed that codon context is even more important than codon usage for translational efficiency [1]. Our findings suggest that certain codon contexts are markedly conserved over all domains of life. However, the comparison of RSCU and RDCU correlations showed that overall codon pair usage is less conserved than single codon usage (Figure 5). This was also confirmed by the shorter branch lengths in the codon pair usage tree than in the single codon usage tree (Figure 6). Tree analysis showed that in the RSCU tree certain phylogenetic classes, for example bacilli, alpha- and gamma-proteobacteria, have extremely similar codon usage preferences within the classes (Figure 6A). However, on the codon pair tree (RDCU tree), species are more distant from each other (codon pair usage is less similar). In contrast, larger phylogenetic groups are positioned together on the RDCU tree. For example, the similarity of codon pair usage in eukaryotes is higher than the similarity of single codon usage (eukaryotes are placed together on the RDCU tree but not on the RSCU tree). The large differences between the RSCU and RDCU tree topologies and branch lengths imply that codon preference and codon pair preference are shaped by different molecular mechanisms.

Codon frequencies correlate with tRNA concentrations, suggesting that this is a major selective force on codon usage patterns [31-33]. The codon pair preferences can be shaped by several different molecular mechanisms. One is the possible decrease of frameshifting errors through avoidance of mononucleotide repeats [8-10]. In addition, it has been suggested that codon context might be influenced by certain structural constraints imposed by two tRNAs occupying the ribosomal P- and A-sites [1,16,23]. Unfortunately, we currently have very limited information about the details of interaction between different tRNAs with the ribosome [29,34,35], which precludes further extension of this hypothesis.

The ribosome contains three sites for tRNA binding: the A-, P- and E-sites. It has been shown that the E-site tRNA can influence decoding in the A-site [24-26]. In addition, it was shown in fungi that the combinations of three consecutive codons are biased and some combinations are even vanished from the ORFeomes [27]. Therefore, bias might also be observed in codon pair usage where codons are separated by three nucleotides (the 1–3 pair). We observed only one conserved 1–3 interaction, over-representation of the pattern GGUnnnGGU, indicating that interactions between the ribosomal E- and A-sites do not influence the codon context as much as interactions between the P- and A-sites. It was shown that the usage of three neighbouring codons is species specific among fungi [27]. Our results correlate with that and suggest that this bias could also be species specific among bacteria and eukaryotes.

## Conclusion

A conserved biased set of codon pairs was found in a dataset covering a large number of organisms from the three domains of life. Most of the pairs had stronger bias on the ORFeome level than on the whole genome level, suggesting that translation has a greater influence on codon pair biases than molecular mechanisms that shape the genomic DNA in general.

## Methods

### Data

We selected 100 bacterial genomes randomly and complemented the random dataset so that all major phylogenetic classes were covered by at least one organism (resulting in 103 bacteria). For archaea we downloaded protein coding sequences for all sequenced genomes (28 genomes, year 2006). In addition, seven eukaryotic genomes were selected – six fungi and human. The protein coding sequences of all bacteria, archaea and fungi were retrieved from . Human protein coding sequences were retrieved from Ensembl [36]. The list of all genomes analyzed and the corresponding accession numbers are provided in [additional file 6].

To compile standardized genomes we randomly selected sequences from the set of all protein coding sequences of the corresponding organism until 150,000 ± 1000 codon pairs were obtained.

### Calculation of observed and expected codon pair counts

For the observed values, we counted the number of all possible sense:sense and sense:stop codon pairs (61 × 64 = 3904 pairs) by computer. The initial expected value of a codon pair was calculated using the frequencies of single codons in protein coding sequences. The expected value for a codon pair in the ORFeome was normalized as previously described [16,17]: the dipeptide bias was removed by multiplying the initial expected value of a codon pair by the normalization coefficient. The normalization coefficient was the ratio of the observed to expected frequencies of the corresponding dipeptide encoded by the codon pair.

To separate translational effects from DNA-related effects influencing codon pair biases we compared the observed/expected ratios of a codon pair in ORFeomes and the corresponding hexanucleotide in genomes. We averaged the observed/expected values of each codon pair over all studied organisms for the comparison of ORFeomes and genomes.

The expected value of a hexanucleotide in a genome was calculated using the frequencies of trinucleotides in genomic sequences of that organism. The trinucleotide frequencies were counted by moving the window one nucleotide at a time. In genomes, the expected values of trinucleotide pairs containing out-frame UAA and UAG triplets on the sense and/or antisense strand were corrected by excluding the frames of coding regions where the given codon pair could not exist. Without normalization the expected values of pairs containing out-frame stops would be exaggerated, so the observed/expected ratio for the given codon pair would also be underestimated.

In each separate ORFeome, only under-represented codon pairs with observed/expected ratios ≤ 0.9 and over-represented codon pairs with observed/expected ratios ≥ 1.1 were subjected to the two-tailed Fisher's exact test. In all analyses, p-values of 0.01 or less were considered statistically significant. We used no multiple correction methods at this point. Codon pairs that were significantly biased in at least 51% of the organisms studied were marked as conserved.

To analyze which nucleotide positions in a codon pair have most influence on biases, we calculated the average observed/expected ratios of all possible sub-patterns covering both adjacent codons (di-, tri, tetra- and pentamers) in ORFeomes over all the organisms studied. The observed and expected values of the sub-patterns were correspondingly summed over all codon pairs that contained the pattern. Among each set of sub-patterns of different lengths, and also for the codon pairs, the z-score was calculated for the observed/expected ratio of each pattern *i*:

$$z_{i}=\frac{\log_{2}{\left(\text{observed/expected}\right)}_{i,n}}{\sigma \left[\log_{2}{\left(\text{observed/expected}\right)}_{n}\right]},$$

where (*observed/expected*)*i*, *n *is the observed/expected ratio of a codon pair or sub-pattern *i *of length *n *and *σ*[log_2_(*observed/expected*)*n*] is the standard deviation of the observed/expected ratios of all sub-patterns of length *n*.

Comparison of the z-scores allowed the most biased nucleotide sub-pattern responsible for the bias of the codon pair to be identified.

The programs for all those calculations were written in Perl.

### Calculation of evolutionary distance and codon context correlation

The bias of single codons was described by relative synonymous codon usage (RSCU). RSCU values are the number of times a particular codon is observed, relative to the number of times that the codon would be observed in the absence of any codon usage bias [37]. To represent the bias of codon pairs, we calculated the relative dicodon usage (RDCU), which was based on the observed/expected ratios of four different sets of codon pairs: 1–2 (neighboring codons), 1–3 (codons separated by one intervening codon), 1–4 (codons separated by two intervening codons) and 1–10 (codons separated by eight intervening codons) as a control. Next, we measured the correlation of RSCU values between each pair of bacteria (Spearman's *ρ*). Similarly, the correlation between RDCU values in pairs of bacteria was calculated. All pairs of bacteria analyzed were divided into nine groups on the basis of the evolutionary distances between them. Pairwise evolutionary distances were retrieved as a 16SRNA distance matrix from the Ribosomal Database Project [28]. Finally, we calculated the average correlation coefficients for each of those groups.

### RSCU and RDCU trees

RSCU and RDCU trees were drawn using the corresponding correlation coefficients calculated previously: the higher the correlation, the shorter the distance between two organisms. For example, the distance between two organisms with identical codon usage would be 0 (1-*ρ*^2 ^with *ρ*^2 ^= 1). Trees were calculated using the Fitch-Margoliash [38] algorithm from PHYLIP software [39] and were edited using TreeDyn software [40].

## Abbreviations

ORF: open reading frame; RSCU: relative synonymous codon usage; RDCU: relative dicodon usage

## Authors' contributions

AT performed the data analysis. TT and MR conceived the study, participated in the study's design, choice of methods and coordination. All authors wrote, read and approved the manuscript.

## Supplementary Material

Additional file 1 **All conserved avoided codon pairs in the organisms studied**. Codon pairs containing out-frame UAA or UAG triplets on the sense and/or antisense strand are shaded blue. The observed/expected ratio in logarithmic scale for each codon pair in the ORFeome and genome is shown. Observed/expected values smaller than -0.58 are shaded green (corresponding to at least a 1.5-fold difference). % – the percentage of organisms in which the codon pair is significantly avoided. A – B – difference between log_2_(obs/exp) ratios in the ORFeome and the genome. A – B < 0 represents a stronger effect on the ORFeome level. The z-score of the most avoided shorter sub-pattern for each codon pair is also shown (shaded yellow). Click here for file

Additional file 2 **All conserved preferred codon pairs in the organisms studied**. Codon pairs containing out-frame UAA or UAG triplets on the sense and/or antisense strand are shaded blue. The observed/expected ratios in logarithmic scale for each codon pair in ORFeome and genome are shown. Observed/expected values greater than 0.58 are shaded green (corresponding to at least a 1.5-fold difference). % – the percentage of organisms in which the codon pair is significantly preferred. A – B – difference between log_2_(obs/exp) ratios in the ORFeome and the genome. A – B > 0 represents a stronger effect on the ORFeome level. The z-score of the most preferred shorter sub-pattern for each codon pair is also shown (shaded yellow). Click here for file

Additional file 3 **The distribution of average observed/expected ratio of patterns of different length in all organisms studied.** Click here for file

Additional file 4 **Avoided codon pairs of different types**. Codon pairs containing out-frame UAA or UAG triplets on the sense and/or antisense strand are shaded blue. The observed/expected ratios in logarithmic scale for each codon pair in ORFeome and genome are shown. Observed/expected values smaller than -0.58 are shaded green (corresponding to at least a 1.5-fold difference). % – the percentage of organisms in which the codon pair is significantly avoided. A – B – difference between log_2_(obs/exp) ratios in the ORFeome and the genome. A – B < 0 represents a stronger effect on the ORFeome level. The z-score of the most avoided shorter sub-pattern for each codon pair is also shown (shaded yellow). For type 1_A _the location of out-frame UAA or UAG is shown by arrows and colors: → (yellow) – on sense strand; ← (blue) – on antisense strand; ↔ (green) – on sense and antisense strands. Click here for file

Additional file 5 **Preferred codon pairs of different types**. Codon pairs containing out-frame UAA or UAG triplets on the sense and/or antisense strand are shaded blue. The observed/expected ratios in logarithmic scale for each codon pair in ORFeome and genome are shown. Observed/expected values greater than 0.58 are shaded green (corresponding to at least a 1.5-fold difference). % – the percentage of organisms in which the codon pair is significantly preferred. A – B – difference between log_2_(obs/exp) ratios in the ORFeome and the genome. A – B > 0 represents a stronger effect on the ORFeome level. The z-score of the most preferred shorter sub-pattern for each codon pair is also shown (shaded yellow). Click here for file

Additional file 6 **List of organisms, genome sequence accession numbers and abbreviations used in Figure**6. Click here for file
