# Supplementary material for: Preferred and avoided codon pairs in three domains of life
Source: BMC Genomics. 2008 Oct 8;9:463. doi: 10.1186/1471-2164-9-463 (PMC2585594; doi:10.1186/1471-2164-9-463)
Supplement: Additional file 1 — All conserved avoided codon pairs in the organisms studied. Codon pairs containing out-frame UAA or UAG triplets on the sense and/or antisense strand are shaded blue. The observed/expected ratio in logarithmic scale for each codon pair in the ORFeome and genome is shown. Observed/expected values smaller than -0.58 are shaded green (corresponding to at least a 1.5-fold difference). % – the percentage of organisms in which the codon pair is significantly avoided. A – B – difference between log2(obs/exp) ratios in the ORFeome and the genome. A – B < 0 represents a stronger effect on the ORFeome level. The z-score of the most avoided shorter sub-pattern for each codon pair is also shown (shaded yellow). [file 1471-2164-9-463-S1.pdf]

1..2↓ codon pairs	%	6mers, log_2(obs/exp) ORFeome (A)	genome (B)	A - B	z-score, ORFeome 6mers	5mers		4mers		3mers		2mers		type
UUCGCA	86	-0.81	-0.86	0.05	-2.32	UUCGnA	-2.88	UnCGnA	-2.55	nnCGCn	-2.52	nnCnnA	-2.04	6_A
GGGGGU	83	-1.12	-0.43	-0.69	-3.30	GGGGGn	-3.49	nnGGGU	-2.20	nnGGGn	-2.20	nnGGnn	-1.34	8_A
UUCGAA	82	-0.76	-0.75	-0.01	-2.25	UUCGnA	-2.88	UnCGnA	-2.55	nnCGnA	-2.36	nnCnnA	-2.04	6_A
CUUAUG	79	-0.92	-0.63	-0.29	-2.70	CUUAnG	-3.71	CUUAnn	-3.68	nUUAnn	-4.26	nnUAnn	-5.11	1_A
GCUAUG	76	-0.76	-0.28	-0.48	-2.25	nCUAUG	-2.98	nCUAnG	-3.95	nCUAnn	-4.45	nnUAnn	-5.11	1_A
ACUAUG	73	-0.71	-0.21	-0.50	-2.10	nCUAUG	-2.98	nCUAnG	-3.95	nCUAnn	-4.45	nnUAnn	-5.11	1_A
GUUAGC	73	-0.92	-0.52	-0.40	-2.70	nUUAGC	-3.49	nUUAGn	-4.23	nnUAGn	-5.42	nnUAnn	-5.11	1_A
CUUAGU	73	-0.94	-0.83	-0.11	-2.78	nUUAGU	-3.18	nUUAGn	-4.23	nnUAGn	-5.42	nnUAnn	-5.11	1_A
UUCGCG	72	-0.84	-0.56	-0.28	-2.47	UnCGCG	-2.88	nnCGCG	-3.41	nnCGCn	-2.52	nnCGnn	-1.81	3_A
GUUAUG	72	-0.71	-0.30	-0.41	-2.03	GUUAnG	-2.69	nUUAnG	-3.55	nUUAnn	-4.26	nnUAnn	-5.11	1_A
CUUACG	72	-0.89	-0.63	-0.26	-2.62	CUUAnG	-3.71	CUUAnn	-3.68	nUUAnn	-4.26	nnUAnn	-5.11	1_A
GGGUAC	71	-0.84	-0.32	-0.52	-2.70	GGGUnC	-2.78	nGGUnC	-2.79	nGGnnC	-2.69	nnGnnC	-2.53	2_A
GACGCA	70	-0.51	-0.31	-0.20	-1.46	nACGCA	-1.89	nnCGCA	-2.09	nnCGCn	-2.52	nnCnnA	-2.04	3_A
GGGUCC	70	-1.22	-1.02	-0.20	-3.59	nGGUCC	-3.71	nGGnCC	-3.41	nnGUCn	-3.02	nnGnnC	-2.53	2_A
AUUAGU	70	-0.69	-0.32	-0.37	-2.03	AUUAGn	-3.18	nUUAGn	-4.23	nnUAGn	-5.42	nnUAnn	-5.11	1_A
GGGGGC	68	-0.79	-0.29	-0.50	-2.25	GGGGGn	-3.49	GGGnnC	-2.67	nGGnnC	-2.69	nnGnnC	-2.53	8_A
AUUAGC	68	-0.84	-0.30	-0.54	-2.47	nUUAGC	-3.49	nUUAGn	-4.23	nnUAGn	-5.42	nnUAnn	-5.11	1_A
GGAGGU	67	-0.42	-0.02	-0.40	-1.11	GGAGGn	-1.81	nGAGGn	-1.65	nGAGnn	-1.73	nnAGnn	-0.88	
UUCGAU	67	-0.47	-0.28	-0.19	-1.34	UUCGAn	-2.06	UUCGnn	-2.43	UnCGnn	-2.20	nnCGnn	-1.81	6_A
GAGGCC	67	-0.51	-0.33	-0.19	-1.46	GnGGCC	-1.98	GnGnCC	-2.55	nnGnCC	-2.52	nnGnnC	-2.53	2_A
AACGCA	67	-0.47	-0.37	-0.10	-1.40	nACGCA	-1.89	nnCGCA	-2.09	nnCGCn	-2.52	nnCnnA	-2.04	3_A
UUCGCU	67	-0.54	-0.44	-0.09	-1.52	UUCGCn	-2.23	UUCGnn	-2.43	nnCGCn	-2.52	nnCGnn	-1.81	6_A
GGUACC	67	-0.74	-0.83	0.10	-2.10	nGUACC	-2.32	nGUACn	-2.20	GnUAnn	-3.02	nnUAnn	-5.11	1_A
GUUAGU	67	-0.81	-0.46	-0.35	-2.39	GUUAGn	-3.28	nUUAGn	-4.23	nnUAGn	-5.42	nnUAnn	-5.11	1_A
AUUAAG	67	-0.67	-0.46	-0.21	-1.90	nUUAAG	-2.69	nUUAnG	-3.55	nUUAnn	-4.26	nnUAnn	-5.11	1_A
GCUAGC	67	-0.92	-0.97	0.05	-2.70	nCUAGC	-3.82	nCUAGn	-4.52	nnUAGn	-5.42	nnUAnn	-5.11	1_A
AUCUUG	66	-0.60	-0.04	-0.56	-1.70	nUCUUG	-1.81	nnCUUG	-1.65	nUCnnG	-1.43	nnCnnG	-1.34	
GGGACC	66	-0.97	-0.83	-0.14	-2.78	GGGnCC	-3.08	nGGnCC	-3.41	nGGnnC	-2.69	nnGnnC	-2.53	2_A
GUGUCA	66	-0.71	-0.67	-0.05	-2.03	GnGUCA	-2.15	GnGUCn	-2.79	nnGUCn	-3.02	nnGUnn	-2.28	
AUCGCA	66	-0.45	-0.46	0.00	-1.22	nUCGCA	-2.15	nUCGnA	-2.43	nnCGCn	-2.52	nnCnnA	-2.04	3_A
AUCUUA	66	-0.62	-0.68	0.06	-1.77	AUCnUA	-1.73	nUCnUA	-1.87	nUCnnA	-1.89	nnCnnA	-2.04	5_A
AUCGCG	65	-0.64	-0.29	-0.34	-1.83	nUCGCG	-2.88	nnCGCG	-3.41	nnCGCn	-2.52	nnCGnn	-1.81	3_A
GGCCAA	65	-0.51	-0.24	-0.27	-1.46	GGCCnA	-1.73	GnCCnA	-1.87	nnCCnA	-2.20	nnCnnA	-2.04	5_A
AACGCG	65	-0.58	-0.31	-0.27	-1.64	AnCGCG	-2.32	nnCGCG	-3.41	nnCGCn	-2.52	nnCGnn	-1.81	3_A
CCUAUG	65	-0.71	-0.43	-0.28	-2.10	nCUAUG	-2.98	nCUAnG	-3.95	nCUAnn	-4.45	nnUAnn	-5.11	1_A
ACUAGC	65	-0.89	-0.68	-0.21	-2.70	nCUAGC	-3.82	nCUAGn	-4.52	nnUAGn	-5.42	nnUAnn	-5.11	1_A
UUUUUU	64	-0.15	0.43	-0.58	-0.40	UUnUUU	-0.44	UUnUnU	-0.45	UnnUnU	-0.55	UnnnnU	-0.66	8_A
UUGGAC	64	-0.74	-0.29	-0.45	-2.10	UUGGnC	-2.32	UnGGnC	-2.09	nnGGnC	-2.52	nnGnnC	-2.53	2_A
GUGUAC	64	-0.76	-0.32	-0.44	-2.17	GnGUAC	-2.32	nnGUAC	-2.55	nnGUAn	-2.36	nnGnnC	-2.53	2_A
UUGGCC	64	-0.60	-0.26	-0.34	-1.64	UUGGnC	-2.32	nnGGCC	-2.43	nnGnCC	-2.52	nnGnnC	-2.53	2_A
GACGCG	64	-0.56	-0.24	-0.31	-1.52	nACGCG	-2.32	nnCGCG	-3.41	nnCGCn	-2.52	nnCGnn	-1.81	3_A
GUCGAA	64	-0.38	-0.10	-0.28	-1.00	nUCGAA	-1.57	nUCGnA	-2.43	nnCGnA	-2.36	nnCnnA	-2.04	5_A
GGAGUC	64	-0.71	-0.49	-0.22	-2.10	nGAGUC	-2.15	nGAGUn	-2.09	nnAGUn	-1.73	nGnnnC	-1.57	
GAGGGU	64	-0.47	-0.30	-0.18	-1.28	GnGGGU	-1.89	nnGGGU	-2.20	nnGGGn	-2.20	nnGGnn	-1.34	7_A
AUUAAU	64	-0.49	0.12	-0.61	-1.40	AUUAAn	-2.50	nUUAAn	-3.41	nUUAnn	-4.26	nnUAnn	-5.11	1_A
AUUAAA	64	-0.43	0.03	-0.46	-1.17	AUUAAn	-2.50	nUUAAn	-3.41	nUUAnn	-4.26	nnUAnn	-5.11	1_A
UUUAGU	64	-0.56	-0.23	-0.33	-1.58	nUUAGU	-3.18	nUUAGn	-4.23	nnUAGn	-5.42	nnUAnn	-5.11	1_A
GUUAAC	64	-0.64	-0.39	-0.25	-1.83	nUUAAC	-2.32	nUUAAn	-3.41	nUUAnn	-4.26	nnUAnn	-5.11	1_A
CUUAAG	64	-0.79	-0.98	0.19	-2.25	CUUAnG	-3.71	CUUAnn	-3.68	nUUAnn	-4.26	nnUAnn	-5.11	1_A
GCGUAC	63	-0.89	-0.30	-0.59	-2.54	nCGUAC	-3.38	nCGUAn	-3.29	nCGUnn	-2.69	nnGnnC	-2.53	2_A
UUCGUG	63	-0.56	-0.27	-0.28	-1.58	UUCGnG	-2.32	nUCGnG	-2.55	nnCGnG	-2.52	nnCGnn	-1.81	6_A
ACGUAC	62	-0.86	-0.27	-0.59	-2.47	nCGUAC	-3.38	nCGUAn	-3.29	nCGUnn	-2.69	nnGnnC	-2.53	2_A
GACGAA	62	-0.29	0.23	-0.51	-0.79	GACGnA	-1.42	nACGnA	-1.76	nnCGnA	-2.36	nnCnnA	-2.04	5_A
CUCCCA	62	-0.74	-0.30	-0.44	-2.17	CUCCnA	-2.32	CUCCnn	-2.43	nnCCnA	-2.20	nnCnnA	-2.04	4_A
GGAGGC	62	-0.56	-0.14	-0.41	-1.58	GGAGnC	-1.89	nGAGnC	-2.09	nGAGnn	-1.73	nGnnnC	-1.57	
GUCCCA	62	-0.60	-0.33	-0.27	-1.70	GUCCnA	-2.23	GUCCnn	-1.98	nnCCnA	-2.20	nnCnnA	-2.04	4_A
GAACGU	62	-0.43	-0.18	-0.26	-1.17	nAACGU	-1.27	nAACnU	-1.34	nnACGn	-1.13	nAnnnU	-0.88	

1..2↓ codon pairs	%	6mers, log_2(obs/exp)		A - B	z-score, ORFeome										type
		ORFeome (A)	genome (B)		6mers	5mers		4mers		3mers		2mers			
CUCGAG	62	-0.69	-0.47	-0.22	-1.97	CUCGnG	-2.23	nUCGnG	-2.55	nnCGnG	-2.52	nnCGnn	-1.81	
UUUAGC	62	-0.74	-0.17	-0.57	-2.17	nUUAGC	-3.49	nUUAGn	-4.23	nnUAGn	-5.42	nnUAnn	-5.11	1_A
AUGUUA	62	-0.54	-0.46	-0.07	-1.52	AUGUnA	-1.50	nUGUnA	-2.43	nnGUnA	-2.52	nnGUnn	-2.28	
GUUACG	62	-0.56	-0.33	-0.23	-1.52	GUUAnG	-2.69	nUUAnG	-3.55	nUUAnn	-4.26	nnUAnn	-5.11	1_A
UUCGGA	62	-0.56	-0.70	0.14	-1.58	UUCGnA	-2.88	UnCGnA	-2.55	nnCGnA	-2.36	nnCnnA	-2.04	6_A
GUUAAA	62	-0.42	-0.16	-0.26	-1.11	GUUAAn	-2.32	nUUAAn	-3.41	nUUAnn	-4.26	nnUAnn	-5.11	1_A
CUUAGC	62	-0.81	-0.69	-0.12	-2.39	nUUAGC	-3.49	nUUAGn	-4.23	nnUAGn	-5.42	nnUAnn	-5.11	1_A
UUCGAG	61	-0.43	-0.17	-0.27	-1.22	UUCGnG	-2.32	nUCGnG	-2.55	nnCGnG	-2.52	nnCGnn	-1.81	6_A
GCUAAG	61	-0.54	-0.68	0.14	-1.52	GCUAnG	-2.69	nCUAnG	-3.95	nCUAnn	-4.45	nnUAnn	-5.11	1_A
CCUAGC	61	-0.92	-1.06	0.14	-2.70	nCUAGC	-3.82	nCUAGn	-4.52	nnUAGn	-5.42	nnUAnn	-5.11	1_A
GUAAUC	60	-0.42	0.30	-0.72	-1.11	nUAAUC	-1.19	nUAAUn	-1.44	nnAnUC	-1.13	nnAnUn	-1.11	
CUCGCG	60	-0.74	-0.36	-0.38	-2.17	nUCGCG	-2.88	nnCGCG	-3.41	nnCGCn	-2.52	nnCGnn	-1.81	3_A
UUGUAC	60	-0.58	-0.22	-0.35	-1.64	UnGUAC	-1.98	nnGUAC	-2.55	nnGUAn	-2.36	nnGnnC	-2.53	2_A
GGGGCC	60	-0.62	-0.60	-0.03	-1.77	GGGnCC	-3.08	nGGnCC	-3.41	nGGnnC	-2.69	nnGnnC	-2.53	8_A
UCAUAU	59	-0.58	-0.23	-0.35	-1.64	UCAUAn	-2.32	nCAUAn	-3.03	UnnUAn	-0.84	nnAnnU	-0.88	9_A
GCAUAU	59	-0.51	-0.22	-0.29	-1.46	nCAUAU	-2.06	nCAUAn	-3.03	nnAUAn	-0.84	nnAnnU	-0.88	9_A
AGAUUC	59	-0.64	-0.41	-0.24	-1.77	AGAUnC	-1.89	nGAnUC	-1.98	nGAnnC	-1.58	nGnnnC	-1.57	
UUCGUA	59	-0.58	-0.36	-0.22	-1.64	UUCGnA	-2.88	UnCGnA	-2.55	nnCGnA	-2.36	nnCnnA	-2.04	6_A
GCAUAC	59	-0.47	-0.25	-0.22	-1.34	GCAUAn	-2.06	nCAUAn	-3.03	nCAnnC	-0.98	nCnnnC	-0.88	9_A
CUGGGU	59	-0.47	-0.27	-0.20	-1.34	CnGGGU	-1.65	nnGGGU	-2.20	nnGGGn	-2.20	nnGGnn	-1.34	7_A
GCUACG	59	-0.60	-0.29	-0.31	-1.70	GCUAnG	-2.69	nCUAnG	-3.95	nCUAnn	-4.45	nnUAnn	-5.11	1_A
AUCGAA	59	-0.36	-0.28	-0.08	-1.00	nUCGAA	-1.57	nUCGnA	-2.43	nnCGnA	-2.36	nnCnnA	-2.04	5_A
AUUAAC	59	-0.62	-0.10	-0.52	-1.77	AUUAAn	-2.50	nUUAAn	-3.41	nUUAnn	-4.26	nnUAnn	-5.11	1_A
UAUAGC	59	-0.51	-0.35	-0.16	-1.46	UnUAGC	-2.88	nnUAGC	-3.81	nnUAGn	-5.42	nnUAnn	-5.11	1_A
CUUACC	59	-0.47	-0.38	-0.09	-1.34	CUUACn	-2.50	CUUAnn	-3.68	nUUAnn	-4.26	nnUAnn	-5.11	1_A
UCUAGC	59	-0.79	-0.76	-0.03	-2.32	nCUAGC	-3.82	nCUAGn	-4.52	nnUAGn	-5.42	nnUAnn	-5.11	1_A
CCUAAG	59	-0.56	-0.98	0.42	-1.58	CCUAnG	-2.78	nCUAnG	-3.95	nCUAnn	-4.45	nnUAnn	-5.11	1_A
GUCGCG	58	-0.60	-0.22	-0.38	-1.70	nUCGCG	-2.88	nnCGCG	-3.41	nnCGCn	-2.52	nnCGnn	-1.81	3_A
UAUAAU	58	-0.45	0.11	-0.56	-1.28	UAUAAn	-1.73	UnUAAn	-2.55	nnUAAn	-3.90	nnUAnn	-5.11	1_A
UAUAGU	58	-0.67	-0.13	-0.54	-1.90	UAUAGn	-2.78	UnUAGn	-3.68	nnUAGn	-5.42	nnUAnn	-5.11	1_A
AGCGCG	58	-0.51	-0.29	-0.23	-1.46	AnCGCG	-2.32	nnCGCG	-3.41	nnCGCn	-2.52	nnCGnn	-1.81	3_A
GGGUUC	58	-0.42	-0.32	-0.10	-1.17	GGGUnC	-2.78	nGGUnC	-2.79	nGGnnC	-2.69	nnGnnC	-2.53	2_A
GCUAGU	58	-0.71	-0.68	-0.03	-2.10	GCUAGn	-3.08	nCUAGn	-4.52	nnUAGn	-5.42	nnUAnn	-5.11	1_A
CUUAAC	58	-0.54	-0.51	-0.03	-1.52	CUUAAn	-2.41	CUUAnn	-3.68	nUUAnn	-4.26	nnUAnn	-5.11	1_A
GGGAAC	57	-0.60	-0.02	-0.58	-1.70	GGGAnC	-2.32	GGGnnC	-2.67	nGGnnC	-2.69	nnGnnC	-2.53	2_A
GCCGCG	57	-0.62	-0.08	-0.54	-1.70	nCCGCG	-2.41	nnCGCG	-3.41	nnCGCn	-2.52	nnCGnn	-1.81	3_A
UACUUG	57	-0.58	-0.04	-0.54	-1.64	nACUUG	-1.65	nnCUUG	-1.65	nnCnUG	-1.13	nnCnnG	-1.34	
CAGAAU	57	-0.47	-0.15	-0.32	-1.34	nAGAAU	-1.42	CnGAAn	-1.34	nnGAAn	-1.13	nnGnAn	-1.11	
UAUAGA	57	-0.67	-0.17	-0.50	-1.83	UAUAGn	-2.78	UnUAGn	-3.68	nnUAGn	-5.42	nnUAnn	-5.11	1_A
GUGGGU	57	-0.42	-0.18	-0.24	-1.11	GnGGGU	-1.89	nnGGGU	-2.20	nnGGGn	-2.20	nnGGnn	-1.34	7_A
GUCCAA	57	-0.51	-0.28	-0.23	-1.46	GUCCnA	-2.23	GUCCnn	-1.98	nnCCnA	-2.20	nnCnnA	-2.04	4_A
GGCGCC	57	-0.67	-0.52	-0.15	-1.90	nGCGCC	-1.50	nGCGCn	-1.98	nnCGCn	-2.52	nnCGnn	-1.81	3_A
UUGGGC	57	-0.51	-0.37	-0.14	-1.40	UUGGnC	-2.32	UnGGnC	-2.09	nnGGnC	-2.52	nnGnnC	-2.53	2_A
GGCCUC	57	-0.42	-0.33	-0.09	-1.58	GGnCUC	-1.65	nGnCUC	-1.54	nnCCUn	-1.43	nGnnnC	-1.57	
GCUCUG	57	-0.49	-0.41	-0.08	-1.40	nCUCUG	-1.98	nCUCUn	-2.09	nCUnnG	-1.58	nnUnnG	-0.88	
GAGACC	57	-0.49	-0.42	-0.07	-1.40	GAGnCC	-1.73	GnGnCC	-2.55	nnGnCC	-2.52	nnGnnC	-2.53	2_A
GAGGGC	57	-0.25	-0.18	-0.07	-0.69	GnGGGC	-1.65	GnGGGn	-2.20	nnGGnC	-2.52	nnGnnC	-2.53	2_A
CUCGCA	57	-0.43	-0.65	0.21	-1.22	nUCGCA	-2.15	nUCGnA	-2.43	nnCGCn	-2.52	nnCnnA	-2.04	3_A
ACUAAC	57	-0.49	-0.47	-0.02	-1.40	ACUAnC	-2.06	ACUAnn	-3.03	nCUAnn	-4.45	nnUAnn	-5.11	1_A
UCUAAG	57	-0.62	-0.88	0.26	-1.77	UCUAnG	-2.32	nCUAnG	-3.95	nCUAnn	-4.45	nnUAnn	-5.11	1_A
AAGGAG	56	-0.34	0.30	-0.64	-0.90	AnGGAG	-1.05	nnGGAG	-1.44	nnGGAn	-1.73	nnGGnn	-1.34	7_A
UCGUAC	56	-0.89	-0.29	-0.60	-2.62	nCGUAC	-3.38	nCGUAn	-3.29	nCGUnn	-2.69	nnGnnC	-2.53	2_A
CUCCAA	56	-0.45	0.08	-0.54	-1.28	CUCCnA	-2.32	CUCCnn	-2.43	nnCCnA	-2.20	nnCnnA	-2.04	4_A
GCGUCC	56	-0.81	-0.38	-0.43	-2.39	GnGUCC	-2.88	nCGUnC	-2.91	nnGUCn	-3.02	nnGnnC	-2.53	2_A
GCAAUC	56	-0.36	-0.15	-0.21	-1.00	nCAAUC	-1.27	nCAnUC	-1.54	nCAnUn	-1.28	nnAnUn	-1.11	
UUGGAA	56	-0.36	-0.16	-0.20	-1.00	UUGGAn	-1.89	UUGGnn	-1.87	nnGGAn	-1.73	nnGGnn	-1.34	7_A
UCUAUG	56	-0.54	-0.22	-0.32	-1.52	nCUAUG	-2.98	nCUAnG	-3.95	nCUAnn	-4.45	nnUAnn	-5.11	1_A

1..2↓ codon pairs	%	6mers, log_2(obs/exp)		A - B	z-score, ORFeome									type
		ORFeome (A)	genome (B)		6mers	5mers		4mers		3mers		2mers		
GGAGCC	56	-0.43	-0.51	0.08	-1.22	GGAGnC	-1.89	nGAGnC	-2.09	nGnnCC	-1.89	nGnnnC	-1.57	
ACUAAG	56	-0.56	-0.86	0.30	-1.58	ACUAnG	-2.69	nCUAnG	-3.95	nCUAnn	-4.45	nnUAnn	-5.11	1_A
GGGUGG	55	-0.60	0.08	-0.68	-1.70	GGGUGn	-1.89	GGGUnn	-1.65	nnGUGn	-1.89	nnGUnn	-2.28	
GCUCUC	55	-0.51	-0.26	-0.26	-1.52	GCUCUn	-1.65	nCUCUn	-2.09	GCnnnC	-0.98	nCnnnC	-0.88	
GGAGUG	55	-0.45	-0.24	-0.22	-1.28	GGAGUn	-1.98	nGAGUn	-2.09	nnAGUn	-1.73	nnAnUn	-1.11	
GUGUCC	55	-0.67	-0.59	-0.08	-1.90	GnGUCC	-2.88	GnGUCn	-2.79	nnGUCn	-3.02	nnGnnC	-2.53	2_A
AUCCCA	55	-0.40	-0.35	-0.05	-1.11	AnCCCA	-1.50	nUCCnA	-1.98	nnCCnA	-2.20	nnCnnA	-2.04	5_A
UUGGAU	55	-0.34	-0.33	-0.01	-0.95	UUGGAn	-1.89	UUGGnn	-1.87	nnGGAn	-1.73	nnGGnn	-1.34	7_A
GCUACC	55	-0.51	-0.32	-0.19	-1.46	GCUAnC	-2.06	nCUAnC	-3.03	nCUAnn	-4.45	nnUAnn	-5.11	1_A
UACGCA	55	-0.43	-0.47	0.03	-1.22	UnCGCA	-2.15	UnCGnA	-2.55	nnCGCn	-2.52	nnCnnA	-2.04	3_A
GUUAAU	55	-0.40	-0.13	-0.27	-1.11	GUUAAn	-2.32	nUUAAn	-3.41	nUUAnn	-4.26	nnUAnn	-5.11	1_A
GUUAAG	55	-0.58	-0.50	-0.08	-1.64	GUUAnG	-2.69	nUUAnG	-3.55	nUUAnn	-4.26	nnUAnn	-5.11	1_A
GAGGAA	54	-0.23	0.37	-0.60	-0.64	GnGGAA	-1.05	GnGGAn	-1.54	nnGGAn	-1.73	nnGGnn	-1.34	7_A
GAGAAU	54	-0.32	0.12	-0.44	-0.90	nAGAAU	-1.42	nnGAAU	-1.23	nnGAAn	-1.13	nnGnAn	-1.11	
GUCCUU	54	-0.40	0.03	-0.43	-1.06	GUCCUn	-1.73	GUCCnn	-1.98	nnCCUn	-1.43	nnCCnn	-1.11	4_A
GCGGCC	54	-0.40	0.02	-0.42	-1.06	GnGGCC	-1.98	GnGnCC	-2.55	nnGnCC	-2.52	nnGnnC	-2.53	2_A
CCGUAC	54	-0.64	-0.23	-0.41	-1.77	nCGUAC	-3.38	nCGUAn	-3.29	nCGUnn	-2.69	nnGnnC	-2.53	2_A
GCGGGC	54	-0.43	-0.03	-0.40	-1.22	nCGGGC	-1.65	GnGGGn	-2.20	nnGGnC	-2.52	nnGnnC	-2.53	2_A
AAGAAU	54	-0.30	0.04	-0.34	-0.84	nAGAAU	-1.42	nnGAAU	-1.23	nnGAAn	-1.13	nnGnAn	-1.11	
ACCGCG	54	-0.51	-0.18	-0.34	-1.46	nCCGCG	-2.41	nnCGCG	-3.41	nnCGCn	-2.52	nnCGnn	-1.81	3_A
AAUUGG	54	-0.58	-0.25	-0.32	-1.64	nAUUGG	-1.81	AnUnGG	-1.87	nnUnGG	-2.36	nnUnGn	-1.81	
ACCCCG	54	-0.58	-0.27	-0.31	-1.64	nCCCCG	-2.41	nCCCCn	-2.43	nnCnCG	-2.04	nnCnCn	-1.34	8_A
GUGGGC	54	-0.42	-0.13	-0.29	-1.17	GnGGGC	-1.65	GnGGGn	-2.20	nnGGnC	-2.52	nnGnnC	-2.53	2_A
CUCUUG	54	-0.34	-0.06	-0.28	-0.95	nUCUUG	-1.81	CUCnnG	-1.65	nUCnnG	-1.43	nnCnnG	-1.34	
AUUACG	54	-0.47	-0.02	-0.45	-1.34	nUUACG	-2.32	nUUAnG	-3.55	nUUAnn	-4.26	nnUAnn	-5.11	1_A
CCUACG	54	-0.76	-0.38	-0.38	-2.17	CCUAnG	-2.78	nCUAnG	-3.95	nCUAnn	-4.45	nnUAnn	-5.11	1_A
GGUAUG	54	-0.38	-0.29	-0.08	-1.06	GnUAUG	-1.89	nnUAUG	-2.67	nnUAnG	-4.08	nnUAnn	-5.11	1_A
GUCUUA	54	-0.47	-0.43	-0.04	-1.34	nUCUUA	-1.73	nUCnUA	-1.87	nUCnnA	-1.89	nnCnnA	-2.04	5_A
CCUACC	54	-0.51	-0.50	-0.01	-1.46	CCUAnC	-2.23	CCUAnn	-3.03	nCUAnn	-4.45	nnUAnn	-5.11	1_A
AUCGGA	54	-0.42	-0.62	0.21	-1.17	AUCGnA	-1.57	nUCGnA	-2.43	nnCGnA	-2.36	nnCnnA	-2.04	5_A
CUUACA	54	-0.49	-0.64	0.15	-1.40	CUUACn	-2.50	CUUAnn	-3.68	nUUAnn	-4.26	nnUAnn	-5.11	1_A
CUUAAU	54	-0.43	-0.46	0.03	-1.22	CUUAAn	-2.41	CUUAnn	-3.68	nUUAnn	-4.26	nnUAnn	-5.11	1_A
GCUAAC	54	-0.45	-0.53	0.08	-1.28	GCUAnC	-2.06	nCUAnC	-3.03	nCUAnn	-4.45	nnUAnn	-5.11	1_A
CUUAAA	54	-0.43	-0.54	0.11	-1.22	CUUAAn	-2.41	CUUAnn	-3.68	nUUAnn	-4.26	nnUAnn	-5.11	1_A
GUCGUA	53	-0.40	0.29	-0.69	-1.11	nUCGUA	-1.89	nUCGnA	-2.43	nnCGnA	-2.36	nnCnnA	-2.04	5_A
GUCCAG	53	-0.40	0.24	-0.64	-1.11	GUCCnG	-2.15	GUCCnn	-1.98	nnCCnG	-1.58	nnCnnG	-1.34	4_A
ACUACG	53	-0.60	0.14	-0.74	-1.70	ACUAnG	-2.69	nCUAnG	-3.95	nCUAnn	-4.45	nnUAnn	-5.11	1_A
GGGGGG	53	-0.58	-0.20	-0.38	-1.64	GGGGGn	-3.49	GnGGGn	-2.20	nnGGGn	-2.20	nnGGnn	-1.34	8_A
UAUAGG	53	-0.86	-0.44	-0.42	-2.47	UAUAGn	-2.78	UnUAGn	-3.68	nnUAGn	-5.42	nnUAnn	-5.11	1_A
CAUAGC	53	-0.56	-0.27	-0.29	-1.58	CnUAGC	-2.98	CnUAGn	-4.23	nnUAGn	-5.42	nnUAnn	-5.11	1_A
GUUACC	53	-0.40	-0.32	-0.08	-1.06	GUUAnC	-1.98	GUUAnn	-3.03	nUUAnn	-4.26	nnUAnn	-5.11	1_A
AUUAGG	53	-0.74	-0.52	-0.22	-2.10	AUUAGn	-3.18	nUUAGn	-4.23	nnUAGn	-5.42	nnUAnn	-5.11	1_A
GCUAAA	53	-0.23	-0.19	-0.04	-0.69	GCUAAn	-1.81	GCUAnn	-2.91	nCUAnn	-4.45	nnUAnn	-5.11	1_A
AUAAUC	52	-0.43	0.33	-0.76	-1.22	AUAAUn	-1.27	nUAAUn	-1.44	nnAnUC	-1.13	nnAnUn	-1.11	
GUCCCG	52	-0.71	-0.24	-0.47	-2.10	GUCCnG	-2.15	GUCCnn	-1.98	nnCnCG	-2.04	nnCnCn	-1.34	4_A
CUCCGU	52	-0.67	-0.21	-0.45	-1.83	CUCCGn	-2.23	CUCCnn	-2.43	CnCCnn	-1.28	nnCCnn	-1.11	4_A
AUUAUA	52	-0.45	0.11	-0.56	-1.28	AUUAnA	-1.81	AUUAnn	-2.79	nUUAnn	-4.26	nnUAnn	-5.11	1_A
ACGGGC	52	-0.47	-0.13	-0.34	-1.34	nCGGGC	-1.65	nCGGnC	-1.65	nnGGnC	-2.52	nnGnnC	-2.53	2_A
AACUUG	52	-0.47	-0.19	-0.28	-1.34	AAnUUG	-1.81	nnCUUG	-1.65	nnCnUG	-1.13	nnCnnG	-1.34	
GUGGCC	52	-0.43	-0.20	-0.23	-1.22	GnGGCC	-1.98	GnGnCC	-2.55	nnGnCC	-2.52	nnGnnC	-2.53	2_A
GUAUAC	52	-0.51	-0.29	-0.22	-1.46	GUAUAn	-1.57	nUAUAn	-1.44	nnAUAn	-0.84	GnnnnC	-0.88	
AUCGUA	52	-0.42	-0.24	-0.17	-1.11	nUCGUA	-1.89	nUCGnA	-2.43	nnCGnA	-2.36	nnCnnA	-2.04	5_A
GGGAUC	52	-0.42	-0.26	-0.16	-1.11	GGGAnC	-2.32	GGGnnC	-2.67	nGGnnC	-2.69	nnGnnC	-2.53	2_A
UCCGUG	52	-0.54	-0.40	-0.14	-1.52	UnCGUG	-1.73	UnCGnG	-2.31	nnCGnG	-2.52	nnCGnn	-1.81	
AUCCAA	52	-0.30	-0.33	0.02	-0.84	AUCCnA	-1.42	nUCCnA	-1.98	nnCCnA	-2.20	nnCnnA	-2.04	5_A
CCUAAC	52	-0.47	-0.67	0.20	-1.34	CCUAnC	-2.23	CCUAnn	-3.03	nCUAnn	-4.45	nnUAnn	-5.11	1_A
CUCCAG	51	-0.58	0.51	-1.09	-1.58	CUCCnG	-2.88	CUCCnn	-2.43	nnCCnG	-1.58	nnCnnG	-1.34	4_A

1..2↓ codon pairs	%	6mers, log_2(obs/exp)		A - B	z-score, ORFeome									type
		ORFeome (A)	genome (B)		6mers	5mers		4mers		3mers		2mers		
CUCCCG	51	-0.97	-0.12	-0.85	-2.87	CUCCnG	-2.88	CUCCnn	-2.43	nnCnCG	-2.04	nnCnCn	-1.34	4_A
GUCCUG	51	-0.64	0.20	-0.84	-1.77	GUCCnG	-2.15	GUCCnn	-1.98	nnCCnG	-1.58	nnCnnG	-1.34	4_A
AGGUAC	51	-0.84	-0.06	-0.77	-2.39	AGGUnC	-2.69	nGGUnC	-2.79	nGGnnC	-2.69	nnGnnC	-2.53	2_A
CUUCUG	51	-0.47	0.28	-0.75	-1.34	CUUnUG	-1.34	CUnCnG	-1.23	nUUnnG	-0.98	nnUnnG	-0.88	
CUCCUU	51	-0.43	0.30	-0.74	-1.17	CUCCUn	-1.98	CUCCnn	-2.43	nnCCUn	-1.43	nnCCnn	-1.11	4_A
GCCCCG	51	-0.60	-0.04	-0.56	-1.64	nCCCCG	-2.41	nCCCCn	-2.43	nnCnCG	-2.04	nnCnCn	-1.34	8_A
CGUAGA	51	-0.71	-0.04	-0.67	-2.03	CGUAGn	-2.98	CnUAGn	-4.23	nnUAGn	-5.42	nnUAnn	-5.11	1_A
CGUAGG	51	-1.06	-0.40	-0.66	-3.12	CnUAGG	-2.98	CnUAGn	-4.23	nnUAGn	-5.42	nnUAnn	-5.11	1_A
AUUGGG	51	-0.45	-0.01	-0.44	-1.28	AUUnGG	-1.57	AnUnGG	-1.87	nnUnGG	-2.36	nnUnGn	-1.81	
ACGGCC	51	-0.42	-0.02	-0.40	-1.11	nCGGCC	-1.81	nnGGCC	-2.43	nnGnCC	-2.52	nnGnnC	-2.53	2_A
GACGCU	51	-0.30	0.01	-0.31	-0.84	GACGCn	-1.50	nACGCn	-1.76	nnCGCn	-2.52	nnCGnn	-1.81	3_A
UCAUAC	51	-0.58	-0.27	-0.31	-1.64	UCAUAn	-2.32	nCAUAn	-3.03	nCAnnC	-0.98	nCnnnC	-0.88	9_A
CCAGUG	51	-0.45	-0.14	-0.31	-1.28	CCAGUn	-1.34	CnAGUn	-1.87	nnAGUn	-1.73	nnAnUn	-1.11	
GUCCUA	51	-0.60	-0.31	-0.29	-1.70	GUCCnA	-2.23	GUCCnn	-1.98	nnCCnA	-2.20	nnCnnA	-2.04	4_A
CUCCCU	51	-0.45	-0.18	-0.27	-1.28	CUCCCn	-2.23	CUCCnn	-2.43	nnCCCn	-1.73	nnCnCn	-1.34	4_A
UCCGCG	51	-0.71	-0.44	-0.27	-2.03	UnCGCG	-2.88	nnCGCG	-3.41	nnCGCn	-2.52	nnCGnn	-1.81	3_A
GGCAAA	51	-0.18	0.07	-0.26	-0.50	GGCnAA	-0.98	GnCnAA	-1.03	nnCnAA	-1.28	nnCnnA	-2.04	5_A
GCGGAA	51	-0.23	0.00	-0.24	-0.64	GnGGAA	-1.05	nCGGAn	-1.54	nCGnAn	-1.73	nnGGnn	-1.34	
ACGGAC	51	-0.43	-0.23	-0.20	-1.22	ACGnAC	-1.73	nCGnAC	-2.55	nnGGnC	-2.52	nnGnnC	-2.53	2_A
CAGGGU	51	-0.43	-0.24	-0.19	-1.22	CnGGGU	-1.65	nnGGGU	-2.20	nnGGGn	-2.20	nnGGnn	-1.34	7_A
ACAAUC	51	-0.30	-0.14	-0.16	-0.84	nCAAUC	-1.27	nCAnUC	-1.54	nCAnUn	-1.28	nnAnUn	-1.11	
GGCCAG	51	-0.29	-0.13	-0.15	-0.79	GGCCnG	-1.42	GnCCnG	-1.44	nnCCnG	-1.58	nnCnnG	-1.34	
GGAUCC	51	-0.71	-0.58	-0.13	-2.10	nGAUCC	-1.73	GGnnCC	-1.98	nGnnCC	-1.89	nGnnnC	-1.57	
ACCCCA	51	-0.45	-0.40	-0.06	-1.28	ACCCCn	-1.98	nCCCCn	-2.43	nnCCnA	-2.20	nnCnnA	-2.04	8_A
GUCGCA	51	-0.42	-0.44	0.02	-1.17	nUCGCA	-2.15	nUCGnA	-2.43	nnCGCn	-2.52	nnCnnA	-2.04	3_A
AUCCUA	51	-0.47	-0.51	0.04	-1.40	nUCCUA	-1.89	nUCCnA	-1.98	nnCCnA	-2.20	nnCnnA	-2.04	5_A
GGUAUA	51	-0.30	-0.38	0.08	-0.84	GnUAUA	-1.19	GGUAnn	-1.87	GnUAnn	-3.02	nnUAnn	-5.11	1_A
GCUAUC	51	-0.30	-0.19	-0.11	-0.84	GCUAnC	-2.06	nCUAnC	-3.03	nCUAnn	-4.45	nnUAnn	-5.11	1_A
GUGUCU	51	-0.49	-0.67	0.17	-1.40	GUGUCn	-2.06	GnGUCn	-2.79	nnGUCn	-3.02	nnGUnn	-2.28	
UGGGCC	51	-0.54	-0.73	0.20	-1.52	nGGGCC	-2.15	nGGnCC	-3.41	nGGnnC	-2.69	nnGnnC	-2.53	2_A
GCUAUA	51	-0.36	-0.35	-0.01	-1.00	GCUAUn	-1.81	GCUAnn	-2.91	nCUAnn	-4.45	nnUAnn	-5.11	1_A
GCUAAU	51	-0.47	-0.32	-0.15	-1.34	GCUAAn	-1.81	GCUAnn	-2.91	nCUAnn	-4.45	nnUAnn	-5.11	1_A
CGUAAG	51	-0.51	-0.66	0.15	-1.46	CGUAnG	-2.32	CnUAnG	-3.68	nnUAnG	-4.08	nnUAnn	-5.11	1_A
ACUAGU	51	-0.79	-0.70	-0.09	-2.25	ACUAGn	-3.38	nCUAGn	-4.52	nnUAGn	-5.42	nnUAnn	-5.11	1_A
UUUAAG	51	-0.51	-0.54	0.03	-1.46	nUUAAG	-2.69	nUUAnG	-3.55	nUUAnn	-4.26	nnUAnn	-5.11	1_A
CCUAGU	51	-0.74	-0.84	0.10	-2.10	CCUAGn	-3.08	nCUAGn	-4.52	nnUAGn	-5.42	nnUAnn	-5.11	1_A
