# Supplementary material for: Preferred and avoided codon pairs in three domains of life
Source: BMC Genomics. 2008 Oct 8;9:463. doi: 10.1186/1471-2164-9-463 (PMC2585594; doi:10.1186/1471-2164-9-463)
Supplement: Additional file 2 — All conserved preferred codon pairs in the organisms studied. Codon pairs containing out-frame UAA or UAG triplets on the sense and/or antisense strand are shaded blue. The observed/expected ratios in logarithmic scale for each codon pair in ORFeome and genome are shown. Observed/expected values greater than 0.58 are shaded green (corresponding to at least a 1.5-fold difference). % – the percentage of organisms in which the codon pair is significantly preferred. A – B – difference between log2(obs/exp) ratios in the ORFeome and the genome. A – B > 0 represents a stronger effect on the ORFeome level. The z-score of the most preferred shorter sub-pattern for each codon pair is also shown (shaded yellow). [file 1471-2164-9-463-S2.pdf]

1..2↑ codon pairs	%	6mers, log_2(obs/exp)		A - B	z-score, ORFeome									type
		ORFeome (A)	genome (B)		6mers	5mers		4mers		3mers		2mers		
GGGCUU	76	0.99	0.44	0.55	3.45	GGGCnU	3.54	GGGCnn	4.11	nnGCUn	3.96	nnGCnn	4.31	1_P
GAGCAG	70	0.51	0.64	-0.13	1.61	GAGCnG	1.98	GnGCnG	2.98	GnGCnn	3.66	nnGCnn	4.31	1_P
GGGCAU	69	0.83	0.39	0.44	2.73	GGGCnU	3.54	GGGCnn	4.11	GnGCnn	3.66	nnGCnn	4.31	1_P
UUUGAA	66	0.31	0.12	0.19	0.98	UnUGAA	0.84	nnUGAA	0.84	nnUGnA	1.17	nnUGnn	1.26	
GACAGC	64	0.70	0.21	0.49	2.28	GACAGn	2.24	GnCAGn	2.39	nnCAGn	2.36	nnCAnn	2.07	2_P
UUUGCC	64	0.51	0.07	0.44	1.61	nUUGCC	1.72	nUUnCC	1.98	nnUGCn	1.65	nnUnCn	2.07	4_P
GGAACA	64	0.82	0.52	0.30	2.62	nGAACA	3.57	nGAAnA	3.76	nnAAnA	3.45	nnAnnA	2.46	
UACAAC	64	0.58	0.36	0.22	1.87	UACAnC	1.85	nACAnC	1.83	nnCAnC	2.13	nnCAnn	2.07	2_P
AUCAUC	64	0.42	0.53	-0.11	1.31	AUCAnC	1.34	nUCAnC	1.90	nnCAnC	2.13	nnCAnn	2.07	2_P
CUUUCU	61	0.91	0.29	0.61	3.06	CUUUCn	2.87	nnUUCU	3.35	nnUUCn	3.86	nnUUnn	2.84	3_P
GUUUCU	61	0.80	0.32	0.48	2.57	nUUUCU	2.83	nnUUCU	3.35	nnUUCn	3.86	nnUUnn	2.84	
GUCAAU	61	0.52	0.31	0.21	1.67	GUCAnU	1.67	GUCAnn	1.61	nUCAnn	1.77	nnCAnn	2.07	2_P
AAAAUA	61	0.72	0.56	0.16	2.33	AnAAUA	2.87	AnAAnA	2.85	nnAAnA	3.45	nnAnnA	2.46	
UUUUUA	61	0.86	0.80	0.06	2.89	nUUUUA	3.05	nUUUnA	3.53	nnUUnA	4.06	nnUUnn	2.84	
AGAAAA	60	1.07	0.57	0.50	3.81	AGAAnA	4.77	nGAAnA	3.76	nnAAnA	3.45	nnAnnA	2.46	
AAUGCC	60	0.38	0.24	0.15	1.21	AnUGCC	1.34	nnUGCC	1.69	nnUGCn	1.65	nnUnCn	2.07	4_P
GCUUAU	59	0.58	-0.12	0.70	1.89	nCUUAU	2.15	nCUUnU	2.92	nCUUnn	3.24	nnUUnn	2.84	
CUUUCA	59	0.74	0.15	0.59	2.36	CUUUCn	2.87	nnUUCA	3.53	nnUUnA	4.06	nnUUnn	2.84	3_P
CUCUAC	59	0.65	0.24	0.41	2.00	CUCUAn	2.52	CnCUAn	2.12	nnCUAn	2.13	nnCnnC	1.87	
GAGCAU	59	0.44	0.23	0.21	1.40	GAGCnU	2.11	GnGCnU	2.79	GnGCnn	3.66	nnGCnn	4.31	1_P
AUUGCC	59	0.34	0.29	0.05	1.08	nUUGCC	1.72	nUUnCC	1.98	nnUGCn	1.65	nnUnCn	2.07	4_P
UUGAUG	59	0.38	0.95	-0.57	1.18	UUGnUG	1.15	UnGnUG	1.31	nnGnUG	1.77	nnGnUn	1.87	
GAAAGC	58	0.54	0.11	0.43	1.70	nAAAGC	1.90	GnAAGn	2.19	nnAAGn	3.24	nnAAnn	1.26	
GAGCUU	58	0.59	0.35	0.24	1.92	GnGCUU	2.28	GnGCUn	2.98	nnGCUn	3.96	nnGCnn	4.31	1_P
GGGCAA	57	0.75	0.51	0.25	2.38	GGGCnA	3.27	GGGCnn	4.11	GnGCnn	3.66	nnGCnn	4.31	1_P
UCUUCU	57	1.19	0.99	0.20	4.47	nCUUCU	4.14	nCUUCn	3.82	nnUUCn	3.86	nnUUnn	2.84	3_P
UUCUUC	57	0.61	0.71	-0.10	1.97	UUCUnC	1.53	UnCUnC	1.61	nnCUnC	1.77	nnCnnC	1.87	
GCUGAA	57	0.31	0.63	-0.32	0.98	GCUGnA	1.34	nCUGnA	1.98	nCUnnA	2.13	nnUGnn	1.26	
GGUGGU	57	0.47	0.77	-0.30	1.49	nGUGGU	1.58	nGUGnU	1.61	nGUnnU	1.77	nnUGnn	1.26	
AUGAGU	56	0.46	0.18	0.28	1.46	AUGAGn	0.89	AnnAGU	0.35	nUGAnn	0.27	AnnAnn<br>AnnnGn<br>nnGnnU	0.00	
CUUUAU	56	0.65	0.37	0.28	2.00	CUUUAn	2.48	CUUUnn	2.32	CnUUnn	2.47	nnUUnn	2.84	3_P
GGCUGG	56	0.44	0.19	0.25	1.37	nGCUGG	1.67	nGCUGn	2.05	nnCUGn	2.58	nnCnGn	1.67	
GCUUCU	55	0.89	0.62	0.27	2.93	nCUUCU	4.14	nCUUCn	3.82	nnUUCn	3.86	nnUUnn	2.84	3_P
CUUUCC	54	0.87	0.18	0.69	2.89	CUUUCn	2.87	CnUUCn	3.10	nnUUCn	3.86	nnUUnn	2.84	3_P
CUCUAU	54	0.57	0.08	0.48	1.84	CUCUAn	2.52	CnCUAn	2.12	nnCUAn	2.13	nnCUnn	1.26	
GGGCAG	54	0.78	0.33	0.45	2.48	GGGCnG	3.19	GGGCnn	4.11	GnGCnn	3.66	nnGCnn	4.31	1_P
ACGCUU	54	0.59	0.15	0.43	1.89	ACGCUn	2.60	nCGCUn	3.17	nnGCUn	3.96	nnGCnn	4.31	1_P
GCCAAU	54	0.44	0.09	0.34	1.37	nCCAAU	1.67	nCCAnU	1.61	nnCAAn	1.77	nnCAnn	2.07	2_P
CUUUAC	54	0.56	0.32	0.25	1.81	CUUUAn	2.48	CUUUnn	2.32	CnUUnn	2.47	nnUUnn	2.84	3_P
AUCAUG	54	0.22	0.00	0.22	0.70	AUCAUn	0.84	AUCAnn	1.16	nUCAnn	1.77	nnCAnn	2.07	2_P
AACUGG	54	0.43	0.30	0.13	1.37	nACUGG	1.63	nACUGn	1.83	nnCUGn	2.58	nnCnGn	1.67	
GGUGCU	54	0.44	0.39	0.04	1.37	nGUGCU	1.63	nGUnCU	2.05	nnUnCU	2.47	nnUnCn	2.07	
GCAACA	54	0.59	0.59	0.00	1.89	nCAACA	2.60	nCAAnA	3.23	nnAAnA	3.45	nnAnnA	2.46	
GAGCGU	54	0.40	0.43	-0.03	1.28	GAGCnU	2.11	GnGCnU	2.79	GnGCnn	3.66	nnGCnn	4.31	1_P
CCUGCU	54	0.65	0.72	-0.06	2.05	nCUGCU	2.44	nCUnCU	3.29	nnUnCU	2.47	nnUnCn	2.07	4_P
AACAGC	54	0.46	0.53	-0.08	1.46	nACAGC	2.15	nACAGn	2.12	nnCAGn	2.36	nnCAnn	2.07	2_P
GGUGUU	54	0.37	0.52	-0.15	1.18	nGUGUU	1.20	nGUGnU	1.61	nGUnnU	1.77	nnUGnn	1.26	
GGAUUG	53	0.67	-0.06	0.73	2.13	nGAUUG	2.20	nnAUUG	1.90	nnAUnG	1.89	nnAUnn	1.06	
CCUUAU	53	0.65	-0.02	0.67	2.03	CCUUAn	2.28	nCUUnU	2.92	nCUUnn	3.24	nnUUnn	2.84	
AGAAGA	53	1.52	0.99	0.53	4.80	AGAAnA	4.77	nGAAnA	3.76	nnAAnA	3.45	nnAnnA	2.46	
GGUUCU	53	0.62	0.17	0.46	1.97	nGUUCU	2.68	nnUUCU	3.35	nnUUCn	3.86	nnUUnn	2.84	4_P
GGGCUG	53	0.76	0.32	0.44	2.41	GGGCnG	3.19	GGGCnn	4.11	nnGCUn	3.96	nnGCnn	4.31	1_P
CUCAAU	53	0.62	0.21	0.40	1.97	CUCAAn	1.76	nnCAAU	1.61	nUCAnn	1.77	nnCAnn	2.07	2_P
GAUUUA	53	0.53	0.14	0.39	1.67	GnUUUA	2.24	nnUUUA	3.29	nnUUnA	4.06	nnUUnn	2.84	
GGAACG	53	0.56	0.17	0.39	1.81	nGAACG	2.03	nGAACn	2.19	nGAAnn	2.01	nnAAnn	1.26	
GGGCUA	53	0.87	0.51	0.35	2.89	GGGCnA	3.27	GGGCnn	4.11	nnGCUn	3.96	nnGCnn	4.31	1_P

1..2↑ codon pairs	%	6mers, log_2(obs/exp)		A - B	z-score, ORFeome									type
		ORFeome (A)	genome (B)		6mers	5mers		4mers		3mers		2mers		
CUUGAA	53	0.33	0.34	-0.01	1.05	CUUGnA	1.10	CUUGnn	1.24	nnUGnA	1.17	nnUGnn	1.26	
CCUGAA	53	0.32	0.65	-0.33	0.98	CCUGnA	1.44	nCUGnA	1.98	nCUnnA	2.13	nnUGnn	1.26	
GCUGCU	53	0.53	0.68	-0.15	1.67	nCUGCU	2.44	nCUnCU	3.29	nnUnCU	2.47	nnUnCn	2.07	4_P
AUCACC	53	0.28	0.56	-0.28	0.88	AUCAnC	1.34	nUCAnC	1.90	nnCAnC	2.13	nnCAnn	2.07	2_P
GCAAGA	52	0.82	0.26	0.56	2.73	nCAAGA	4.41	nCAnGA	3.94	nnAAnA	3.45	nnAnnA	2.46	
UUGCUU	52	0.50	0.04	0.46	1.58	UUGCUn	2.07	nnGCUU	2.66	nnGCUn	3.96	nnGCnn	4.31	1_P
GUUGCU	52	0.43	0.24	0.18	1.34	GUUnCU	1.85	nUUnCU	2.05	nnUnCU	2.47	nnUnCn	2.07	4_P
CUUGAU	52	0.37	0.46	-0.09	1.18	CUUGnU	1.15	CUUGnn	1.24	nnUGnU	1.17	nnUGnn	1.26	
GUAUUU	52	0.49	0.41	0.07	1.55	nUAUUU	1.98	nUAUUn	2.05	nUAUnn	2.47	nnAUnn	1.06	
GAUGCC	52	0.25	0.18	0.07	0.81	nAUGCC	1.25	nnUGCC	1.69	nnUGCn	1.65	nnUnCn	2.07	4_P
UUGCUG	52	0.56	0.60	-0.04	1.81	UUGCUn	2.07	nnGCUG	2.72	nnGCUn	3.96	nnGCnn	4.31	1_P
AUCAAC	52	0.30	0.42	-0.12	0.95	nUCAAC	1.39	nUCAnC	1.90	nnCAnC	2.13	nnCAnn	2.07	2_P
GUGAUG	52	0.39	0.93	-0.54	1.21	GUGnUG	1.49	GnGnUG	1.61	nnGnUG	1.77	nnGnUn	1.87	
GCAAGG	51	0.78	0.28	0.49	2.45	nCAAGG	3.27	nCAAGn	2.66	nnAAGn	3.24	nnAAnn	1.26	
AGUGAA	51	0.35	0.23	0.12	1.12	AGUGnA	1.15	AGUnnA	1.16	nnUGnA	1.17	nnUGnn	1.26	
CCUUCU	51	0.98	0.69	0.29	3.32	nCUUCU	4.14	nCUUCn	3.82	nnUUCn	3.86	nnUUnn	2.84	3_P
GCUUCA	51	0.69	0.44	0.24	2.23	nCUUCA	4.08	nCUUnA	3.99	nnUUnA	4.06	nnUUnn	2.84	3_P
ACCAAU	51	0.33	0.09	0.24	1.05	nCCAAU	1.67	nCCAnU	1.61	nnCAAn	1.77	nnCAnn	2.07	2_P
GCGCUU	51	0.41	0.19	0.22	1.28	GnGCUU	2.28	nCGCUn	3.17	nnGCUn	3.96	nnGCnn	4.31	1_P
GAUGUG	51	0.21	0.02	0.20	0.70	nAUGUG	0.74	nAUGUn	0.68	GnUGnn	0.79	nnUGnn	1.26	
CUGCAA	51	0.51	0.34	0.17	1.61	CnGCAA	1.72	CnGCnA	2.25	CnGCnn	3.13	nnGCnn	4.31	1_P
AAAACA	51	0.46	0.44	0.02	1.46	AnAACA	2.44	nnAACA	2.92	nnAAnA	3.45	nnAnnA	2.46	
AUUUUA	51	0.60	0.65	-0.05	1.92	nUUUUA	3.05	nUUUnA	3.53	nnUUnA	4.06	nnUUnn	2.84	
GAGCAA	51	0.33	0.38	-0.05	1.05	GAGCnA	1.67	GAGCnn	2.52	GnGCnn	3.66	nnGCnn	4.31	1_P
GUGCUG	51	0.51	0.75	-0.24	1.64	GnGCUG	2.15	GnGCUn	2.98	nnGCUn	3.96	nnGCnn	4.31	1_P
