# Supplementary figures and images for: Preferred and avoided codon pairs in three domains of life
Source: BMC Genomics. 2008 Oct 8;9:463. doi: 10.1186/1471-2164-9-463 (PMC2585594; doi:10.1186/1471-2164-9-463)

**The distribution of average obs/exp ratio in all studied genomes**

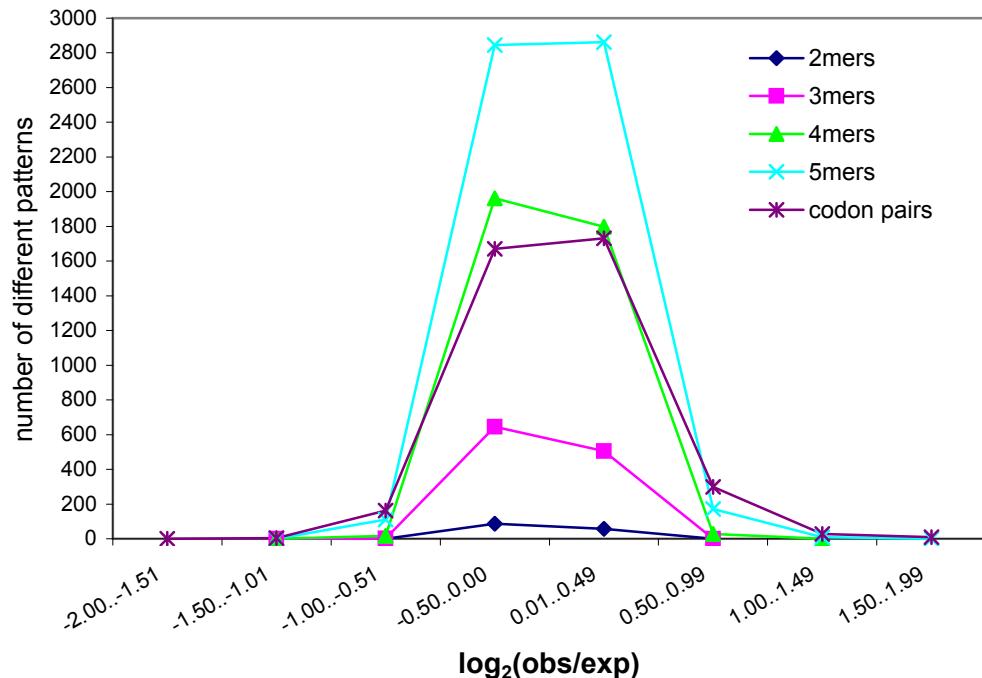

Supplement: Additional file 3 — The distribution of average observed/expected ratio of patterns of different length in all organisms studied. [file 1471-2164-9-463-S3.pdf]
