# Supplementary material for: Preferred and avoided codon pairs in three domains of life
Source: BMC Genomics. 2008 Oct 8;9:463. doi: 10.1186/1471-2164-9-463 (PMC2585594; doi:10.1186/1471-2164-9-463)
Supplement: Additional file 4 — Avoided codon pairs of different types. Codon pairs containing out-frame UAA or UAG triplets on the sense and/or antisense strand are shaded blue. The observed/expected ratios in logarithmic scale for each codon pair in ORFeome and genome are shown. Observed/expected values smaller than -0.58 are shaded green (corresponding to at least a 1.5-fold difference). % – the percentage of organisms in which the codon pair is significantly avoided. A – B – difference between log2(obs/exp) ratios in the ORFeome and the genome. A – B < 0 represents a stronger effect on the ORFeome level. The z-score of the most avoided shorter sub-pattern for each codon pair is also shown (shaded yellow). For type 1A the location of out-frame UAA or UAG is shown by arrows and colors: → (yellow) – on sense strand; ← (blue) – on antisense strand; ↔ (green) – on sense and antisense strands. [file 1471-2164-9-463-S4.pdf]

type 1 nn**UA**nn

1..2↓ codon pairs	strand	%	6mers, log_2(obs/exp) ORFeome (A)	genome (B)	A - B	z-score, ORFeome 6mers	5mers		4mers		3mers		2mers	
ACUACG	←	53	-0.60	0.14	-0.74	-1.70	ACUAnG	-2.69	nCUAnG	-3.95	nCUAnn	-4.45	nnUAnn	-5.11
CGUAGA	→	51	-0.71	-0.04	-0.68	-2.03	CGUAGn	-2.98	CnUAGn	-4.23	nnUAGn	-5.42	nnUAnn	-5.11
CGUAGG	→	51	-1.06	-0.40	-0.66	-3.12	CnUAGG	-2.98	CnUAGn	-4.23	nnUAGn	-5.42	nnUAnn	-5.11
AUUAAU	↔	64	-0.49	0.12	-0.61	-1.40	AUUAAn	-2.50	nUUAAn	-3.41	nUUAnn	-4.26	nnUAnn	-5.11
UAUAAU	→	58	-0.45	0.11	-0.57	-1.28	UAUAAn	-1.73	UnUAAn	-2.55	nnUAAn	-3.90	nnUAnn	-5.11
UUUAGC	↔	62	-0.74	-0.17	-0.57	-2.17	nUUAGC	-3.49	nUUAGn	-4.23	nnUAGn	-5.42	nnUAnn	-5.11
AUUAUA	←	52	-0.45	0.11	-0.56	-1.28	AUUAnA	-1.81	AUUAnn	-2.79	nUUAnn	-4.26	nnUAnn	-5.11
AUUAGC	↔	68	-0.84	-0.30	-0.54	-2.47	nUUAGC	-3.49	nUUAGn	-4.23	nnUAGn	-5.42	nnUAnn	-5.11
UAUAGU	→	58	-0.67	-0.13	-0.53	-1.90	UAUAGn	-2.78	UnUAGn	-3.68	nnUAGn	-5.42	nnUAnn	-5.11
AUUAAC	↔	59	-0.62	-0.10	-0.52	-1.77	AUUAAn	-2.50	nUUAAn	-3.41	nUUAnn	-4.26	nnUAnn	-5.11
ACUAUG	←	73	-0.71	-0.21	-0.50	-2.10	nCUAUG	-2.98	nCUAnG	-3.95	nCUAnn	-4.45	nnUAnn	-5.11
UAUAGA	→	57	-0.67	-0.17	-0.49	-1.83	UAUAGn	-2.78	UnUAGn	-3.68	nnUAGn	-5.42	nnUAnn	-5.11
GCUAUG	←	76	-0.76	-0.28	-0.48	-2.25	nCUAUG	-2.98	nCUAnG	-3.95	nCUAnn	-4.45	nnUAnn	-5.11
AUUAAA	↔	64	-0.43	0.03	-0.46	-1.17	AUUAAn	-2.50	nUUAAn	-3.41	nUUAnn	-4.26	nnUAnn	-5.11
AUUACG	←	54	-0.47	-0.02	-0.45	-1.34	nUUACG	-2.32	nUUAnG	-3.55	nUUAnn	-4.26	nnUAnn	-5.11
UAUAGG	→	53	-0.86	-0.44	-0.43	-2.47	UAUAGn	-2.78	UnUAGn	-3.68	nnUAGn	-5.42	nnUAnn	-5.11
GUUAUG	←	72	-0.71	-0.30	-0.41	-2.03	GUUAnG	-2.69	nUUAnG	-3.55	nUUAnn	-4.26	nnUAnn	-5.11
GUUAGC	↔	73	-0.92	-0.52	-0.39	-2.70	nUUAGC	-3.49	nUUAGn	-4.23	nnUAGn	-5.42	nnUAnn	-5.11
CCUACG	←	54	-0.76	-0.38	-0.38	-2.17	CCUAnG	-2.78	nCUAnG	-3.95	nCUAnn	-4.45	nnUAnn	-5.11
AUUAGU	↔	70	-0.69	-0.32	-0.37	-2.03	AUUAGn	-3.18	nUUAGn	-4.23	nnUAGn	-5.42	nnUAnn	-5.11
GUUAGU	↔	67	-0.81	-0.46	-0.36	-2.39	GUUAGn	-3.28	nUUAGn	-4.23	nnUAGn	-5.42	nnUAnn	-5.11
UUUAGU	↔	64	-0.56	-0.23	-0.32	-1.58	nUUAGU	-3.18	nUUAGn	-4.23	nnUAGn	-5.42	nnUAnn	-5.11
UCUAUG	←	56	-0.54	-0.22	-0.32	-1.52	nCUAUG	-2.98	nCUAnG	-3.95	nCUAnn	-4.45	nnUAnn	-5.11
GCUACG	←	59	-0.60	-0.29	-0.31	-1.70	GCUAnG	-2.69	nCUAnG	-3.95	nCUAnn	-4.45	nnUAnn	-5.11
CUUAUG	←	79	-0.92	-0.63	-0.29	-2.70	CUUAnG	-3.71	CUUAnn	-3.68	nUUAnn	-4.26	nnUAnn	-5.11
CAUAGC	→	53	-0.56	-0.27	-0.29	-1.58	CnUAGC	-2.98	CnUAGn	-4.23	nnUAGn	-5.42	nnUAnn	-5.11
CCUAUG	←	65	-0.71	-0.43	-0.28	-2.10	nCUAUG	-2.98	nCUAnG	-3.95	nCUAnn	-4.45	nnUAnn	-5.11
GUUAAU	↔	55	-0.40	-0.13	-0.27	-1.11	GUUAAn	-2.32	nUUAAn	-3.41	nUUAnn	-4.26	nnUAnn	-5.11
CUUACG	←	72	-0.89	-0.63	-0.26	-2.62	CUUAnG	-3.71	CUUAnn	-3.68	nUUAnn	-4.26	nnUAnn	-5.11
GUUAAA	↔	62	-0.42	-0.16	-0.25	-1.11	GUUAAn	-2.32	nUUAAn	-3.41	nUUAnn	-4.26	nnUAnn	-5.11
GUUAAC	↔	64	-0.64	-0.39	-0.25	-1.83	nUUAAC	-2.32	nUUAAn	-3.41	nUUAnn	-4.26	nnUAnn	-5.11
GUUACG	←	62	-0.56	-0.33	-0.23	-1.52	GUUAnG	-2.69	nUUAnG	-3.55	nUUAnn	-4.26	nnUAnn	-5.11
AUUAGG	↔	53	-0.74	-0.52	-0.22	-2.10	AUUAGn	-3.18	nUUAGn	-4.23	nnUAGn	-5.42	nnUAnn	-5.11
ACUAGC	↔	65	-0.89	-0.68	-0.21	-2.70	nCUAGC	-3.82	nCUAGn	-4.52	nnUAGn	-5.42	nnUAnn	-5.11
AUUAAG	↔	67	-0.67	-0.46	-0.20	-1.90	nUUAAG	-2.69	nUUAnG	-3.55	nUUAnn	-4.26	nnUAnn	-5.11
GCUACC	←	55	-0.51	-0.32	-0.20	-1.46	GCUAnC	-2.06	nCUAnC	-3.03	nCUAnn	-4.45	nnUAnn	-5.11
UAUAGC	→	59	-0.51	-0.35	-0.16	-1.46	UnUAGC	-2.88	nnUAGC	-3.81	nnUAGn	-5.42	nnUAnn	-5.11
GCUAAU	↔	51	-0.47	-0.32	-0.16	-1.34	GCUAAn	-1.81	GCUAnn	-2.91	nCUAnn	-4.45	nnUAnn	-5.11
CUUAGC	↔	62	-0.81	-0.69	-0.12	-2.39	nUUAGC	-3.49	nUUAGn	-4.23	nnUAGn	-5.42	nnUAnn	-5.11
GCUAUC	←	51	-0.30	-0.19	-0.11	-0.84	GCUAnC	-2.06	nCUAnC	-3.03	nCUAnn	-4.45	nnUAnn	-5.11
CUUAGU	↔	73	-0.94	-0.83	-0.11	-2.78	nUUAGU	-3.18	nUUAGn	-4.23	nnUAGn	-5.42	nnUAnn	-5.11
CUUACC	←	59	-0.47	-0.38	-0.09	-1.34	CUUACn	-2.50	CUUAnn	-3.68	nUUAnn	-4.26	nnUAnn	-5.11
ACUAGU	↔	51	-0.79	-0.70	-0.09	-2.25	ACUAGn	-3.38	nCUAGn	-4.52	nnUAGn	-5.42	nnUAnn	-5.11
GGUAUG		54	-0.38	-0.29	-0.08	-1.06	GnUAUG	-1.89	nnUAUG	-2.67	nnUAnG	-4.08	nnUAnn	-5.11
GUUAAG	↔	55	-0.58	-0.50	-0.08	-1.64	GUUAnG	-2.69	nUUAnG	-3.55	nUUAnn	-4.26	nnUAnn	-5.11
GUUACC	←	53	-0.40	-0.32	-0.08	-1.06	GUUAnC	-1.98	GUUAnn	-3.03	nUUAnn	-4.26	nnUAnn	-5.11
GCUAAA	↔	53	-0.23	-0.19	-0.05	-0.69	GCUAAn	-1.81	GCUAnn	-2.91	nCUAnn	-4.45	nnUAnn	-5.11
GCUAGU	↔	58	-0.71	-0.68	-0.04	-2.10	GCUAGn	-3.08	nCUAGn	-4.52	nnUAGn	-5.42	nnUAnn	-5.11
CUUAAC	↔	58	-0.54	-0.51	-0.03	-1.52	CUUAAn	-2.41	CUUAnn	-3.68	nUUAnn	-4.26	nnUAnn	-5.11
UCUAGC	↔	59	-0.79	-0.76	-0.03	-2.32	nCUAGC	-3.82	nCUAGn	-4.52	nnUAGn	-5.42	nnUAnn	-5.11
ACUAAC	↔	57	-0.49	-0.47	-0.02	-1.40	ACUAnC	-2.06	ACUAnn	-3.03	nCUAnn	-4.45	nnUAnn	-5.11
CCUACC	←	54	-0.51	-0.50	-0.02	-1.46	CCUAnC	-2.23	CCUAnn	-3.03	nCUAnn	-4.45	nnUAnn	-5.11
GCUAUA	←	51	-0.36	-0.35	-0.01	-1.00	GCUAUn	-1.81	GCUAnn	-2.91	nCUAnn	-4.45	nnUAnn	-5.11
UUUAAG	↔	51	-0.51	-0.54	0.02	-1.46	nUUAAG	-2.69	nUUAnG	-3.55	nUUAnn	-4.26	nnUAnn	-5.11
CUUAAU	↔	54	-0.43	-0.46	0.03	-1.22	CUUAAn	-2.41	CUUAnn	-3.68	nUUAnn	-4.26	nnUAnn	-5.11
GCUAGC	↔	67	-0.92	-0.97	0.05	-2.70	nCUAGC	-3.82	nCUAGn	-4.52	nnUAGn	-5.42	nnUAnn	-5.11
GCUAAC	↔	54	-0.45	-0.53	0.07	-1.28	GCUAnC	-2.06	nCUAnC	-3.03	nCUAnn	-4.45	nnUAnn	-5.11

type 1 nn**UA**nn (continues)

1..2↓ codon pairs	strand	%	6mers, log_2(obs/exp) ORFeome (A)	genome (B)	A - B	z-score, ORFeome 6mers	5mers		4mers		3mers		2mers	
GGUAUA		51	-0.30	-0.38	0.08	-0.84	GnUAUA	-1.19	GGUAnn	-1.87	GnUAnn	-3.02	nnUAnn	-5.11
GGUACC		67	-0.74	-0.83	0.10	-2.10	nGUACC	-2.32	nGUACn	-2.20	GnUAnn	-3.02	nnUAnn	-5.11
CCUAGU	↔	51	-0.74	-0.84	0.10	-2.10	CCUAGn	-3.08	nCUAGn	-4.52	nnUAGn	-5.42	nnUAnn	-5.11
CUUAAA	↔	54	-0.43	-0.54	0.10	-1.22	CUUAAn	-2.41	CUUAnn	-3.68	nUUAnn	-4.26	nnUAnn	-5.11
GCUAAG	↔	61	-0.54	-0.68	0.14	-1.52	GCUAnG	-2.69	nCUAnG	-3.95	nCUAnn	-4.45	nnUAnn	-5.11
CUUACA	←	54	-0.49	-0.64	0.14	-1.40	CUUACn	-2.50	CUUAnn	-3.68	nUUAnn	-4.26	nnUAnn	-5.11
CCUAGC	↔	61	-0.92	-1.06	0.14	-2.70	nCUAGC	-3.82	nCUAGn	-4.52	nnUAGn	-5.42	nnUAnn	-5.11
CGUAAG	→	51	-0.51	-0.66	0.15	-1.46	CGUAnG	-2.32	CnUAnG	-3.68	nnUAnG	-4.08	nnUAnn	-5.11
CUUAAG	↔	64	-0.79	-0.98	0.19	-2.25	CUUAnG	-3.71	CUUAnn	-3.68	nUUAnn	-4.26	nnUAnn	-5.11
CCUAAC	↔	52	-0.47	-0.67	0.19	-1.34	CCUAnC	-2.23	CCUAnn	-3.03	nCUAnn	-4.45	nnUAnn	-5.11
UCUAAG	↔	57	-0.62	-0.88	0.26	-1.77	UCUAnG	-2.32	nCUAnG	-3.95	nCUAnn	-4.45	nnUAnn	-5.11
ACUAAG	↔	56	-0.56	-0.86	0.30	-1.58	ACUAnG	-2.69	nCUAnG	-3.95	nCUAnn	-4.45	nnUAnn	-5.11
CCUAAG	↔	59	-0.56	-0.98	0.42	-1.58	CCUAnG	-2.78	nCUAnG	-3.95	nCUAnn	-4.45	nnUAnn	-5.11

		%
none		4.29
sense	→	12.86
antisense	←	28.57
both	↔	54.29

type 2 nn**G**nn**C**

1..2↓ codon pairs	%	6mers, log_2(obs/exp)		A - B	z-score, ORFeome								
		ORFeome (A)	genome (B)		6mers	5mers		4mers		3mers		2mers	
AGGUAC	51	-0.84	-0.06	-0.77	-2.39	AGGUnC	-2.69	nGGUnC	-2.79	nGGnnC	-2.69	nnGnnC	-2.53
UCGUAC	56	-0.89	-0.29	-0.60	-2.62	nCGUAC	-3.38	nCGUAn	-3.29	nCGUnn	-2.69	nnGnnC	-2.53
ACGUAC	62	-0.86	-0.27	-0.59	-2.47	nCGUAC	-3.38	nCGUAn	-3.29	nCGUnn	-2.69	nnGnnC	-2.53
GCGUAC	63	-0.89	-0.30	-0.59	-2.54	nCGUAC	-3.38	nCGUAn	-3.29	nCGUnn	-2.69	nnGnnC	-2.53
GGGAAC	57	-0.60	-0.02	-0.58	-1.70	GGGAnC	-2.32	GGGnnC	-2.67	nGGnnC	-2.69	nnGnnC	-2.53
GGGUAC	71	-0.84	-0.32	-0.52	-2.70	GGGUnC	-2.78	nGGUnC	-2.79	nGGnnC	-2.69	nnGnnC	-2.53
UUGGAC	64	-0.74	-0.29	-0.45	-2.10	UUGGnC	-2.32	UnGGnC	-2.09	nnGGnC	-2.52	nnGnnC	-2.53
GUGUAC	64	-0.76	-0.32	-0.44	-2.17	GnGUAC	-2.32	nnGUAC	-2.55	nnGUAn	-2.36	nnGnnC	-2.53
GCGUCC	56	-0.81	-0.38	-0.43	-2.39	GnGUCC	-2.88	nCGUnC	-2.91	nnGUCn	-3.02	nnGnnC	-2.53
GCGGCC	54	-0.40	0.02	-0.42	-1.06	GnGGCC	-1.98	GnGnCC	-2.55	nnGnCC	-2.52	nnGnnC	-2.53
CCGUAC	54	-0.64	-0.23	-0.41	-1.77	nCGUAC	-3.38	nCGUAn	-3.29	nCGUnn	-2.69	nnGnnC	-2.53
GCGGGC	54	-0.43	-0.03	-0.40	-1.22	nCGGGC	-1.65	GnGGGn	-2.20	nnGGnC	-2.52	nnGnnC	-2.53
ACGGCC	51	-0.42	-0.02	-0.40	-1.11	nCGGCC	-1.81	nnGGCC	-2.43	nnGnCC	-2.52	nnGnnC	-2.53
UUGUAC	60	-0.58	-0.22	-0.35	-1.64	UnGUAC	-1.98	nnGUAC	-2.55	nnGUAn	-2.36	nnGnnC	-2.53
ACGGGC	52	-0.47	-0.13	-0.34	-1.34	nCGGGC	-1.65	nCGGnC	-1.65	nnGGnC	-2.52	nnGnnC	-2.53
UUGGCC	64	-0.60	-0.26	-0.34	-1.64	UUGGnC	-2.32	nnGGCC	-2.43	nnGnCC	-2.52	nnGnnC	-2.53
GUGGGC	54	-0.42	-0.13	-0.29	-1.17	GnGGGC	-1.65	GnGGGn	-2.20	nnGGnC	-2.52	nnGnnC	-2.53
GUGGCC	52	-0.43	-0.20	-0.23	-1.22	GnGGCC	-1.98	GnGnCC	-2.55	nnGnCC	-2.52	nnGnnC	-2.53
ACGGAC	51	-0.43	-0.23	-0.20	-1.22	ACGnAC	-1.73	nCGnAC	-2.55	nnGGnC	-2.52	nnGnnC	-2.53
GGGUCC	70	-1.22	-1.02	-0.20	-3.59	nGGUCC	-3.71	nGGnCC	-3.41	nnGUCn	-3.02	nnGnnC	-2.53
GAGGCC	67	-0.51	-0.33	-0.19	-1.46	GnGGCC	-1.98	GnGnCC	-2.55	nnGnCC	-2.52	nnGnnC	-2.53
GGGAUC	52	-0.42	-0.26	-0.16	-1.11	GGGAnC	-2.32	GGGnnC	-2.67	nGGnnC	-2.69	nnGnnC	-2.53
UUGGGC	57	-0.51	-0.37	-0.14	-1.40	UUGGnC	-2.32	UnGGnC	-2.09	nnGGnC	-2.52	nnGnnC	-2.53
GGGACC	66	-0.97	-0.83	-0.14	-2.78	GGGnCC	-3.08	nGGnCC	-3.41	nGGnnC	-2.69	nnGnnC	-2.53
GGGUUC	58	-0.42	-0.32	-0.10	-1.17	GGGUnC	-2.78	nGGUnC	-2.79	nGGnnC	-2.69	nnGnnC	-2.53
GUGUCC	55	-0.67	-0.59	-0.08	-1.90	GnGUCC	-2.88	GnGUCn	-2.79	nnGUCn	-3.02	nnGnnC	-2.53
GAGACC	57	-0.49	-0.42	-0.07	-1.40	GAGnCC	-1.73	GnGnCC	-2.55	nnGnCC	-2.52	nnGnnC	-2.53
GAGGGC	57	-0.25	-0.18	-0.07	-0.69	GnGGGC	-1.65	GnGGGn	-2.20	nnGGnC	-2.52	nnGnnC	-2.53
GGGGCC	60	-0.62	-0.60	-0.03	-1.77	GGGnCC	-3.08	nGGnCC	-3.41	nGGnnC	-2.69	nnGnnC	-2.53

type 3 nn**CGC**n

1..2↓ codon pairs	%	6mers, log_2(obs/exp)		A - B	z-score, ORFeome								
		ORFeome (A)	genome (B)		6mers	5mers		4mers		3mers		2mers	
GCCGCG	57	-0.62	-0.08	-0.54	-1.70	nCCGCG	-2.41	nnCGCG	-3.41	nnCGCn	-2.52	nnCGnn	-1.81
CUCGCG	60	-0.74	-0.36	-0.38	-2.17	nUCGCG	-2.88	nnCGCG	-3.41	nnCGCn	-2.52	nnCGnn	-1.81
GUCGCG	58	-0.60	-0.22	-0.38	-1.70	nUCGCG	-2.88	nnCGCG	-3.41	nnCGCn	-2.52	nnCGnn	-1.81
AUCGCG	65	-0.64	-0.29	-0.34	-1.83	nUCGCG	-2.88	nnCGCG	-3.41	nnCGCn	-2.52	nnCGnn	-1.81
ACCGCG	54	-0.51	-0.18	-0.34	-1.46	nCCGCG	-2.41	nnCGCG	-3.41	nnCGCn	-2.52	nnCGnn	-1.81
GACGCG	64	-0.56	-0.24	-0.31	-1.52	nACGCG	-2.32	nnCGCG	-3.41	nnCGCn	-2.52	nnCGnn	-1.81
GACGCU	51	-0.30	0.01	-0.31	-0.84	GACGCn	-1.50	nACGCn	-1.76	nnCGCn	-2.52	nnCGnn	-1.81
UUCGCG	72	-0.84	-0.56	-0.28	-2.47	UnCGCG	-2.88	nnCGCG	-3.41	nnCGCn	-2.52	nnCGnn	-1.81
AACGCG	65	-0.58	-0.31	-0.27	-1.64	AnCGCG	-2.32	nnCGCG	-3.41	nnCGCn	-2.52	nnCGnn	-1.81
UCCGCG	51	-0.71	-0.44	-0.27	-2.03	UnCGCG	-2.88	nnCGCG	-3.41	nnCGCn	-2.52	nnCGnn	-1.81
AGCGCG	58	-0.51	-0.29	-0.23	-1.46	AnCGCG	-2.32	nnCGCG	-3.41	nnCGCn	-2.52	nnCGnn	-1.81
GACGCA	70	-0.51	-0.31	-0.20	-1.46	nACGCA	-1.89	nnCGCA	-2.09	nnCGCn	-2.52	nnCnnA	-2.04
GGCGCC	57	-0.67	-0.52	-0.15	-1.90	nGCGCC	-1.50	nGCGCn	-1.98	nnCGCn	-2.52	nnCGnn	-1.81
AACGCA	67	-0.47	-0.37	-0.10	-1.40	nACGCA	-1.89	nnCGCA	-2.09	nnCGCn	-2.52	nnCnnA	-2.04
AUCGCA	66	-0.45	-0.46	0.00	-1.22	nUCGCA	-2.15	nUCGnA	-2.43	nnCGCn	-2.52	nnCnnA	-2.04
GUCGCA	51	-0.42	-0.44	0.02	-1.17	nUCGCA	-2.15	nUCGnA	-2.43	nnCGCn	-2.52	nnCnnA	-2.04
UACGCA	55	-0.43	-0.47	0.03	-1.22	UnCGCA	-2.15	UnCGnA	-2.55	nnCGCn	-2.52	nnCnnA	-2.04
CUCGCA	57	-0.43	-0.65	0.21	-1.22	nUCGCA	-2.15	nUCGnA	-2.43	nnCGCn	-2.52	nnCnnA	-2.04

type 4 **(G/C)UCC**nn

1..2↓ codon pairs	%	6mers, log_2(obs/exp)		A - B	z-score, ORFeome								
		ORFeome (A)	genome (B)		6mers	5mers		4mers		3mers		2mers	
CUCCAG	51	-0.58	0.51	-1.09	-1.58	CUCCnG	-2.88	CUCCnn	-2.43	nnCCnG	-1.58	nnCnnG	-1.34
CUCCCG	51	-0.97	-0.12	-0.85	-2.87	CUCCnG	-2.88	CUCCnn	-2.43	nnCnCG	-2.04	nnCnCn	-1.34
GUCCUG	51	-0.64	0.20	-0.84	-1.77	GUCCnG	-2.15	GUCCnn	-1.98	nnCCnG	-1.58	nnCnnG	-1.34
CUCCUU	51	-0.43	0.30	-0.74	-1.17	CUCCUn	-1.98	CUCCnn	-2.43	nnCCUn	-1.43	nnCCnn	-1.11
GUCCAG	53	-0.40	0.24	-0.64	-1.11	GUCCnG	-2.15	GUCCnn	-1.98	nnCCnG	-1.58	nnCnnG	-1.34
CUCCAA	56	-0.45	0.08	-0.54	-1.28	CUCCnA	-2.32	CUCCnn	-2.43	nnCCnA	-2.20	nnCnnA	-2.04
GUCCCG	52	-0.71	-0.24	-0.47	-2.10	GUCCnG	-2.15	GUCCnn	-1.98	nnCnCG	-2.04	nnCnCn	-1.34
CUCCGU	52	-0.67	-0.21	-0.45	-1.83	CUCCGn	-2.23	CUCCnn	-2.43	CnCCnn	-1.28	nnCCnn	-1.11
CUCCCA	62	-0.74	-0.30	-0.44	-2.17	CUCCnA	-2.32	CUCCnn	-2.43	nnCCnA	-2.20	nnCnnA	-2.04
GUCCUU	54	-0.40	0.03	-0.43	-1.06	GUCCUn	-1.73	GUCCnn	-1.98	nnCCUn	-1.43	nnCCnn	-1.11
GUCCUA	51	-0.60	-0.31	-0.29	-1.70	GUCCnA	-2.23	GUCCnn	-1.98	nnCCnA	-2.20	nnCnnA	-2.04
CUCCCU	51	-0.45	-0.18	-0.27	-1.28	CUCCCn	-2.23	CUCCnn	-2.43	nnCCCn	-1.73	nnCnCn	-1.34
GUCCCA	62	-0.60	-0.33	-0.27	-1.70	GUCCnA	-2.23	GUCCnn	-1.98	nnCCnA	-2.20	nnCnnA	-2.04
GUCCAA	57	-0.51	-0.28	-0.23	-1.46	GUCCnA	-2.23	GUCCnn	-1.98	nnCCnA	-2.20	nnCnnA	-2.04

type 5 nn**C**nn**A**

1..2↓ codon pairs	%	6mers, log_2(obs/exp)		A - B	z-score, ORFeome								
		ORFeome (A)	genome (B)		6mers	5mers		4mers		3mers		2mers	
GUCGUA	53	-0.40	0.29	-0.69	-1.11	nUCGUA	-1.89	nUCGnA	-2.43	nnCGnA	-2.36	nnCnnA	-2.04
GACGAA	62	-0.29	0.23	-0.51	-0.79	GACGnA	-1.42	nACGnA	-1.76	nnCGnA	-2.36	nnCnnA	-2.04
GUCGAA	64	-0.38	-0.10	-0.28	-1.00	nUCGAA	-1.57	nUCGnA	-2.43	nnCGnA	-2.36	nnCnnA	-2.04
GGCCAA	65	-0.51	-0.24	-0.27	-1.46	GGCCnA	-1.73	GnCCnA	-1.87	nnCCnA	-2.20	nnCnnA	-2.04
GGCAAA	51	-0.18	0.07	-0.26	-0.50	GGCnAA	-0.98	GnCnAA	-1.03	nnCnAA	-1.28	nnCnnA	-2.04
AUCGUA	52	-0.42	-0.24	-0.17	-1.11	nUCGUA	-1.89	nUCGnA	-2.43	nnCGnA	-2.36	nnCnnA	-2.04
AUCGAA	59	-0.36	-0.28	-0.08	-1.00	nUCGAA	-1.57	nUCGnA	-2.43	nnCGnA	-2.36	nnCnnA	-2.04
AUCCCA	55	-0.40	-0.35	-0.05	-1.11	AnCCCA	-1.50	nUCCnA	-1.98	nnCCnA	-2.20	nnCnnA	-2.04
GUCUUA	54	-0.47	-0.43	-0.04	-1.34	nUCUUA	-1.73	nUCnUA	-1.87	nUCnnA	-1.89	nnCnnA	-2.04
AUCCAA	52	-0.30	-0.33	0.02	-0.84	AUCCnA	-1.42	nUCCnA	-1.98	nnCCnA	-2.20	nnCnnA	-2.04
AUCCUA	51	-0.47	-0.51	0.04	-1.40	nUCCUA	-1.89	nUCCnA	-1.98	nnCCnA	-2.20	nnCnnA	-2.04
AUCUUA	66	-0.62	-0.68	0.06	-1.77	AUCnUA	-1.73	nUCnUA	-1.87	nUCnnA	-1.89	nnCnnA	-2.04
AUCGGA	54	-0.42	-0.62	0.21	-1.17	AUCGnA	-1.57	nUCGnA	-2.43	nnCGnA	-2.36	nnCnnA	-2.04

type 6 **UUCG**nn

1..2↓ codon pairs	%	6mers, log_2(obs/exp)		A - B	z-score, ORFeome								
		ORFeome (A)	genome (B)		6mers	5mers		4mers		3mers		2mers	
UUCGUG	63	-0.56	-0.27	-0.28	-1.58	UUCGnG	-2.32	nUCGnG	-2.55	nnCGnG	-2.52	nnCGnn	-1.81
UUCGAG	61	-0.43	-0.17	-0.27	-1.22	UUCGnG	-2.32	nUCGnG	-2.55	nnCGnG	-2.52	nnCGnn	-1.81
UUCGUA	59	-0.58	-0.36	-0.22	-1.64	UUCGnA	-2.88	UnCGnA	-2.55	nnCGnA	-2.36	nnCnnA	-2.04
UUCGAU	67	-0.47	-0.28	-0.19	-1.34	UUCGAn	-2.06	UUCGnn	-2.43	UnCGnn	-2.20	nnCGnn	-1.81
UUCGCU	67	-0.54	-0.44	-0.09	-1.52	UUCGCn	-2.23	UUCGnn	-2.43	nnCGCn	-2.52	nnCGnn	-1.81
UUCGAA	82	-0.76	-0.75	-0.01	-2.25	UUCGnA	-2.88	UnCGnA	-2.55	nnCGnA	-2.36	nnCnnA	-2.04
UUCGCA	86	-0.81	-0.86	0.05	-2.32	UUCGnA	-2.88	UnCGnA	-2.55	nnCGCn	-2.52	nnCnnA	-2.04
UUCGGA	62	-0.56	-0.70	0.14	-1.58	UUCGnA	-2.88	UnCGnA	-2.55	nnCGnA	-2.36	nnCnnA	-2.04

type 7 nn**GG**nn

1..2↓ codon pairs	%	6mers, log_2(obs/exp)		A - B	z-score, ORFeome								
		ORFeome (A)	genome (B)		6mers	5mers		4mers		3mers		2mers	
AAGGAG	56	-0.34	0.30	-0.64	-0.90	AnGGAG	-1.05	nnGGAG	-1.44	nnGGAn	-1.73	nnGGnn	-1.34
GAGGAA	54	-0.23	0.37	-0.60	-0.64	GnGGAA	-1.05	GnGGAn	-1.54	nnGGAn	-1.73	nnGGnn	-1.34
GUGGGU	57	-0.42	-0.18	-0.24	-1.11	GnGGGU	-1.89	nnGGGU	-2.20	nnGGGn	-2.20	nnGGnn	-1.34
CUGGGU	59	-0.47	-0.27	-0.20	-1.34	CnGGGU	-1.65	nnGGGU	-2.20	nnGGGn	-2.20	nnGGnn	-1.34
UUGGAA	56	-0.36	-0.16	-0.20	-1.00	UUGGAn	-1.89	UUGGnn	-1.87	nnGGAn	-1.73	nnGGnn	-1.34
CAGGGU	51	-0.43	-0.24	-0.19	-1.22	CnGGGU	-1.65	nnGGGU	-2.20	nnGGGn	-2.20	nnGGnn	-1.34
GAGGGU	64	-0.47	-0.30	-0.18	-1.28	GnGGGU	-1.89	nnGGGU	-2.20	nnGGGn	-2.20	nnGGnn	-1.34
UUGGAU	55	-0.34	-0.33	-0.01	-0.95	UUGGAn	-1.89	UUGGnn	-1.87	nnGGAn	-1.73	nnGGnn	-1.34

type 8 **mononucleotide repeats**

1..2↓ codon pairs	%	6mers, log$_2$(obs/exp) ORFeome (A)	6mers, log$_2$(obs/exp) genome (B)	A - B	z-score, ORFeome 6mers	5mers		4mers		3mers		2mers	
GGGGGU	83	-1.12	-0.43	-0.69	-3.30	GGGGGn	-3.49	nnGGGU	-2.20	nnGGGn	-2.20	nnGGnn	-1.34
UUUUUU	64	-0.15	0.43	-0.58	-0.40	UUnUUU	-0.44	UUnUnU	-0.45	UnnUnU	-0.55	UnnnnU	-0.66
GCCCCG	51	-0.60	-0.04	-0.56	-1.64	nCCCCG	-2.41	nCCCCn	-2.43	nnCnCG	-2.04	nnCnCn	-1.34
GGGGGC	68	-0.79	-0.29	-0.50	-2.25	GGGGGn	-3.49	GGGnnC	-2.67	nGGnnC	-2.69	nnGnnC	-2.53
GGGGGG	53	-0.58	-0.20	-0.38	-1.64	GGGGGn	-3.49	GnGGGn	-2.20	nnGGGn	-2.20	nnGGnn	-1.34
ACCCCG	54	-0.58	-0.27	-0.31	-1.64	nCCCCG	-2.41	nCCCCn	-2.43	nnCnCG	-2.04	nnCnCn	-1.34
ACCCCA	51	-0.45	-0.40	-0.06	-1.28	ACCCCn	-1.98	nCCCCn	-2.43	nnCCnA	-2.20	nnCnnA	-2.04
GGGGCC	60	-0.62	-0.60	-0.03	-1.77	GGGnCC	-3.08	nGGnCC	-3.41	nGGnnC	-2.69	nnGnnC	-2.53

type 9 n**CAUA**n

1..2↓ codon pairs	%	6mers, log$_2$(obs/exp) ORFeome (A)	6mers, log$_2$(obs/exp) genome (B)	A - B	z-score, ORFeome 6mers	5mers		4mers		3mers		2mers	
UCAUAU	59	-0.58	-0.23	-0.35	-1.64	UCAUAn	-2.32	nCAUAn	-3.03	UnnUAn	-0.84	nnAnnU	-0.88
UCAUAC	51	-0.58	-0.27	-0.31	-1.64	UCAUAn	-2.32	nCAUAn	-3.03	nCAnnC	-0.98	nCnnnC	-0.88
GCAUAU	59	-0.51	-0.22	-0.29	-1.46	nCAUAU	-2.06	nCAUAn	-3.03	nnAUAn	-0.84	nnAnnU	-0.88
GCAUAC	59	-0.47	-0.25	-0.22	-1.34	GCAUAn	-2.06	nCAUAn	-3.03	nCAnnC	-0.98	nCnnnC	-0.88
