# Supplementary material for: Preferred and avoided codon pairs in three domains of life
Source: BMC Genomics. 2008 Oct 8;9:463. doi: 10.1186/1471-2164-9-463 (PMC2585594; doi:10.1186/1471-2164-9-463)
Supplement: Additional file 5 — Preferred codon pairs of different types. Codon pairs containing out-frame UAA or UAG triplets on the sense and/or antisense strand are shaded blue. The observed/expected ratios in logarithmic scale for each codon pair in ORFeome and genome are shown. Observed/expected values greater than 0.58 are shaded green (corresponding to at least a 1.5-fold difference). % – the percentage of organisms in which the codon pair is significantly preferred. A – B – difference between log2(obs/exp) ratios in the ORFeome and the genome. A – B > 0 represents a stronger effect on the ORFeome level. The z-score of the most preferred shorter sub-pattern for each codon pair is also shown (shaded yellow). [file 1471-2164-9-463-S5.pdf]

type 1 nn**GC**nn

1..2↑ codon pairs	%	6mers, log$_2$(obs/exp)		A - B	z-score, ORFeome								
		ORFs (A)	genome (B)		6mers	5mers		4mers		3mers		2mers	
GGGCUU	76	0.99	0.44	0.55	3.45	GGGCnU	3.54	GGGCnn	4.11	nnGCUn	3.96	nnGCnn	4.31
UUGCUU	52	0.50	0.04	0.46	1.58	UUGCUn	2.07	nnGCUU	2.66	nnGCUn	3.96	nnGCnn	4.31
GGGCAG	54	0.78	0.33	0.45	2.48	GGGCnG	3.19	GGGCnn	4.11	GnGCnn	3.66	nnGCnn	4.31
GGGCAU	69	0.83	0.39	0.44	2.73	GGGCnU	3.54	GGGCnn	4.11	GnGCnn	3.66	nnGCnn	4.31
GGGCUG	53	0.76	0.32	0.44	2.41	GGGCnG	3.19	GGGCnn	4.11	nnGCUn	3.96	nnGCnn	4.31
ACGCUU	54	0.59	0.15	0.43	1.89	ACGCUn	2.60	nCGCUn	3.17	nnGCUn	3.96	nnGCnn	4.31
GGGCUA	53	0.87	0.51	0.35	2.89	GGGCnA	3.27	GGGCnn	4.11	nnGCUn	3.96	nnGCnn	4.31
GGGCAA	57	0.75	0.51	0.25	2.38	GGGCnA	3.27	GGGCnn	4.11	GnGCnn	3.66	nnGCnn	4.31
GAGCUU	58	0.59	0.35	0.24	1.92	GnGCUU	2.28	GnGCUn	2.98	nnGCUn	3.96	nnGCnn	4.31
GCGCUU	51	0.41	0.19	0.22	1.28	GnGCUU	2.28	nCGCUn	3.17	nnGCUn	3.96	nnGCnn	4.31
GAGCAU	59	0.44	0.23	0.21	1.40	GAGCnU	2.11	GnGCnU	2.79	GnGCnn	3.66	nnGCnn	4.31
CUGCAA	51	0.51	0.34	0.17	1.61	CnGCAA	1.72	CnGCnA	2.25	CnGCnn	3.13	nnGCnn	4.31
GAGCGU	54	0.40	0.43	-0.03	1.28	GAGCnU	2.11	GnGCnU	2.79	GnGCnn	3.66	nnGCnn	4.31
UUGCUG	52	0.56	0.60	-0.04	1.81	UUGCUn	2.07	nnGCUG	2.72	nnGCUn	3.96	nnGCnn	4.31
GAGCAA	51	0.33	0.38	-0.05	1.05	GAGCnA	1.67	GAGCnn	2.52	GnGCnn	3.66	nnGCnn	4.31
GAGCAG	70	0.51	0.64	-0.13	1.61	GAGCnG	1.98	GnGCnG	2.98	GnGCnn	3.66	nnGCnn	4.31
GUGCUG	51	0.51	0.75	-0.24	1.64	GnGCUG	2.15	GnGCUn	2.98	nnGCUn	3.96	nnGCnn	4.31

type 2 nn**CA**nn

1..2↑ codon pairs	%	6mers, log$_2$(obs/exp)		A - B	z-score, ORFeome								
		ORFs (A)	genome (B)		6mers	5mers		4mers		3mers		2mers	
GACAGC	64	0.70	0.21	0.49	2.28	GACAGn	2.24	GnCAGn	2.39	nnCAGn	2.36	nnCAnn	2.07
CUCAAU	53	0.62	0.21	0.40	1.97	CUCAAn	1.76	nnCAAU	1.61	nUCAnn	1.77	nnCAnn	2.07
GCCAAU	54	0.44	0.09	0.34	1.37	nCCAAU	1.67	nCCAnU	1.61	nnCAAn	1.77	nnCAnn	2.07
ACCAAU	51	0.33	0.09	0.24	1.05	nCCAAU	1.67	nCCAnU	1.61	nnCAAn	1.77	nnCAnn	2.07
AUCAUG	54	0.22	0.00	0.22	0.70	AUCAUn	0.84	AUCAnn	1.16	nUCAnn	1.77	nnCAnn	2.07
UACAAC	64	0.58	0.36	0.22	1.87	UACAnC	1.85	nACAnC	1.83	nnCAnC	2.13	nnCAnn	2.07
GUCAAU	61	0.52	0.31	0.21	1.67	GUCAnU	1.67	GUCAnn	1.61	nUCAnn	1.77	nnCAnn	2.07
AACAGC	54	0.46	0.53	-0.08	1.46	nACAGC	2.15	nACAGn	2.12	nnCAGn	2.36	nnCAnn	2.07
AUCAUC	64	0.42	0.53	-0.11	1.31	AUCAnC	1.34	nUCAnC	1.90	nnCAnC	2.13	nnCAnn	2.07
AUCAAC	52	0.30	0.42	-0.12	0.95	nUCAAC	1.39	nUCAnC	1.90	nnCAnC	2.13	nnCAnn	2.07
AUCACC	53	0.28	0.56	-0.28	0.88	AUCAnC	1.34	nUCAnC	1.90	nnCAnC	2.13	nnCAnn	2.07

type 3 nn**UU**nn

1..2↑ codon pairs	%	6mers, log$_2$(obs/exp)		A - B	z-score, ORFeome								
		ORFs (A)	genome (B)		6mers	5mers		4mers		3mers		2mers	
CUUUCC	54	0.87	0.18	0.69	2.89	CUUUCn	2.87	CnUUCn	3.10	nnUUCn	3.86	nnUUnn	2.84
CUUUCU	61	0.91	0.29	0.61	3.06	CUUUCn	2.87	nnUUCU	3.35	nnUUCn	3.86	nnUUnn	2.84
CUUUCA	59	0.74	0.15	0.59	2.36	CUUUCn	2.87	nnUUCA	3.53	nnUUnA	4.06	nnUUnn	2.84
CCUUCU	51	0.98	0.69	0.29	3.32	nCUUCU	4.14	nCUUCn	3.82	nnUUCn	3.86	nnUUnn	2.84
CUUUAU	56	0.65	0.37	0.28	2.00	CUUUAn	2.48	CUUUnn	2.32	CnUUnn	2.47	nnUUnn	2.84
GCUUCU	55	0.89	0.62	0.27	2.93	nCUUCU	4.14	nCUUCn	3.82	nnUUCn	3.86	nnUUnn	2.84
CUUUAC	54	0.56	0.32	0.25	1.81	CUUUAn	2.48	CUUUnn	2.32	CnUUnn	2.47	nnUUnn	2.84
GCUUCA	51	0.69	0.44	0.24	2.23	nCUUCA	4.08	nCUUnA	3.99	nnUUnA	4.06	nnUUnn	2.84
UCUUCU	57	1.19	0.99	0.20	4.47	nCUUCU	4.14	nCUUCn	3.82	nnUUCn	3.86	nnUUnn	2.84

type 4 nn**U**n**C**n

1..2↑ codon pairs	%	6mers, log$_2$(obs/exp)		A - B	z-score, ORFeome									
		ORFs (A)	genome (B)		6mers	5mers		4mers		3mers		2mers		
GGUUCU	53	0.62	0.17	0.46	1.97	nGUUCU	2.68	nnUUCU	3.35	nnUUCn	3.86	nnUUnn	2.84	
UUUGCC	64	0.51	0.07	0.44	1.61	nUUGCC	1.72	nUUnCC	1.98	nnUGCn	1.65	nnUnCn	2.07	
GUUGCU	52	0.43	0.24	0.18	1.34	GUUnCU	1.85	nUUnCU	2.05	nnUnCU	2.47	nnUnCn	2.07	
AAUGCC	60	0.38	0.24	0.15	1.21	AnUGCC	1.34	nnUGCC	1.69	nnUGCn	1.65	nnUnCn	2.07	
GAUGCC	52	0.25	0.18	0.07	0.81	nAUGCC	1.25	nnUGCC	1.69	nnUGCn	1.65	nnUnCn	2.07	
AUUGCC	59	0.34	0.29	0.05	1.08	nUUGCC	1.72	nUUnCC	1.98	nnUGCn	1.65	nnUnCn	2.07	
CCUGCU	54	0.65	0.72	-0.06	2.05	nCUGCU	2.44	nCUnCU	3.29	nnUnCU	2.47	nnUnCn	2.07	
GCUGCU	53	0.53	0.68	-0.15	1.67	nCUGCU	2.44	nCUnCU	3.29	nnUnCU	2.47	nnUnCn	2.07	
