# Supplementary material for: Preferred and avoided codon pairs in three domains of life
Source: BMC Genomics. 2008 Oct 8;9:463. doi: 10.1186/1471-2164-9-463 (PMC2585594; doi:10.1186/1471-2164-9-463)
Supplement: Additional file 6 — List of organisms, genome sequence accession numbers and abbreviations used in Figure6. [file 1471-2164-9-463-S6.pdf]

BACTERIA (Refseq 27.09.2006)	Abbreviation	Accession no
Acidobacteria bacterium Ellin345	Abact	NC_008009
Agrobacterium tumefaciens str. C58_UWash	Atume	NC_003304, NC_003305
Anabaena variabilis ATCC 29413	Avari	NC_007413
Aquifex aeolicus VF5	Aaeol	NC_000918
Bacillus anthracis str. Ames	Bant1	NC_003997
Bacillus anthracis str. Sterne	Bant2	NC_005945
Bacillus cereus ATCC 10987	Bcer1	NC_003909
Bacillus cereus ATCC 14579	Bcer2	NC_004722
Bacillus clausii KSM-K16	Bclau	NC_006582
Bacillus halodurans C-125	Bhalo	NC_002570
Bacillus licheniformis ATCC 14580	Blich	NC_006270
Bacteroides fragilis YCH46	Bfrag	NC_006347
Bacteroides thetaiotaomicron VPI-5482	Bthet	NC_004663
Bartonella quintana str. Toulouse	Bquin	NC_005955
Bifidobacterium longum NCC2705	Blong	NC_004307
Brucella abortus biovar 1 str. 9-941	Babor	NC_006932, NC_006933
Brucella melitensis biovar Abortus 2308	Bmeli	NC_007618
Buchnera aphidicola str. Sg	Baphi	NC_004061
Burkholderia pseudomallei 1710b	Bpse1	NC_007434, NC_007435
Burkholderia pseudomallei K96243	Bpse2	NC_006350, NC_006351
Burkholderia sp. 383	Burk	NC_007509, NC_007510, NC_007511
Burkholderia thailandensis E264	Bthai	NC_007650, NC_007651
Burkholderia xenovorans LB400	Bxeno	NC_007951, NC_007952, NC_007953
Campylobacter jejuni RM1221	Cjej1	NC_003912
Campylobacter jejuni subsp. Jejuni NCTC 11168	Cjej2	NC_002163
Candidatus blochmannia pennsylvanicus str. BPEN	Cbloc	NC_007292
Chlamydia trachomatis D/UW-3/CX	Ctrac	NC_000117
Chromobacterium violaceum ATCC 12472	Cviol	NC_005085
Chromohalobacter salexigens DSM 3043	Csale	NC_007963
Corynebacterium glutamicum ATCC 13032	Cglut	NC_003450
Corynebacterium jeikeium K411	Cjeik	NC_007164
Dechloromonas aromatica RCB	Darom	NC_007298
Dehalococcoides ethenogens 195	Dethe	NC_002936
Ehrlichia canis str. Jake	Ecani	NC_007354
Escherichia coli K-12	Ecoli	NC_000913
Francisella tularensis subsp. Tularensis FSC198	Ftula	NC_008245
Frankia alni ACN14a	Falni	NC_008278
Fusobacterium nucleatum subsp. nucleatum ATCC 25586	Fnucl	NC_003454
Haemophilus ducrey 35000HP	Hducr	NC_002940
Haemophilus influenzae 86-028NP	Hinfl	NC_007146
Hahella chejuensis KCTC 2396	Hchej	NC_007645
Helicobacter hepaticus ATCC 51449	Hhepa	NC_004917
Helicobacter pylori 26695	Hpylo	NC_000915
Idiomarina loihiensis L2TR	Iloih	NC_006512
Jannaschia sp. CCS1	Janna	NC_007802
Lactobacillus delbrueckii subsp. Bulgaricus ATCC 11842	Ldelb	NC_008054
Lactobacillus sakei subsp. sakei 23K	Lsake	NC_007576
Legionella pneumophila str. Lens	Lpne1	NC_006369
Legionella pneumophila str. Paris	Lpne2	NC_006368
Leifsonia xyli subsp.xyli str. CTCB07	Lxyli	NC_006087
Leptospira interrogans serovar Lai str.56601	Linte	NC_004342, NC_004343
Magnetospirillum magneticum AMB-1	Mmagn	NC_007626
Mesorhizobium loti MAFF303099	Mloti	NC_002678

BACTERIA (Refseq 27.09.2006)	Abbreviation	Accession no
Mesorhizobium sp. BNC1	Meso	NC_008254
Methylococcus capsulatus str. Bath	Mcaps	NC_002977
Mycoplasma capricolum subsp. Capricolum ATCC 27343	Mcapr	NC_007633
Mycoplasma hyopneumoniae 7448	Mhyop	NC_007332
Mycoplasma penetrans HF-2	Mpene	NC_004432
Mycoplasma pneumoniae M129	Mpneu	NC_000912
Nitrobacter hamburgensis X14	Mhamb	NC_007964
Pseudoalteromonas haloplanktis TAC125	Phalo	NC_007481, NC_007482
Pseudomonas putida KT2440	Pputi	NC_002947
Pseudomonas syringae pv. Syringae B728a	Psyr1	NC_007005
Pseudomonas syringae pv. tomato str. DC3000	Psyr2	NC_004578
Psychrobacter arcticus 273-4	Parct	NC_007204
Rhodococcus sp. RHA1	Rhodo	NC_008268
Rhodoferax ferrireducens T118	Rferr	NC_007908
Rhodopirellula baltica	Rbalt	NC_005027
Rhodopseudomonas palustris BisB18	Rpalu	NC_007925
Rickettsia felis URRWXCal2	Rfeli	NC_007109
Rickettsia typhi str. Wilmington	Rtyph	NC_006142
Salmonella enterica subsp. enterica serovar Paratyphi A str. ATCC 9150	Sente	NC_006511
Shigella flexneri 2a str. 2457T	Sfle1	NC_004741
Shigella flexneri 2a str. 301	Sfle2	NC_004337
Shigella sonnei Ss046	Ssonn	NC_007384
Sodalis glossinidus str. 'morsitans'	Sglos	NC_007712
Staphylococcus aureus RF122	Saur1	NC_007622
Staphylococcus aureus subsp. aureus 8325	Saur2	NC_007795
Staphylococcus aureus subsp. aureus MRSA252	Saur3	NC_002952
Staphylococcus aureus subsp. aureus N315	Saur4	NC_002745
Staphylococcus epidermidis ATCC 12228	Sepi1	NC_004461
Staphylococcus epidermidis RP62A	Sepi2	NC_002976
Staphylococcus saprophyticus subsp. saprophyticus ATCC 15305	Ssapr	NC_007350
Streptococcus agalactiae A909	Sagal	NC_007432
Streptococcus mutans UA 159	Smuta	NC_004350
Streptococcus pyogenes MGAS10270	Spyo1	NC_008022
Streptococcus pyogenes MGAS2096	Spyo2	NC_008023
Streptococcus pyogenes MGAS5005	Spyo3	NC_007297
Streptococcus pyogenes MGAS9429	Spyo4	NC_008021
Synechococcus CC9311	Syne1	NC_008319
Synechococcus CC9605	Syne2	NC_007516
Synthrophus aciditrophicus SB	Sacid1	NC_007759
Thermosynechococcus elongatus BP-1	Telon	NC_004113
Thermotoga maritima MSB8	Tmari	NC_000853
Thermus termophilus H27	Tterm1	NC_005835
Thermus termophilus HB8	Tterm2	NC_006461
Trichodesmium erythraeum IMS101	Teryt	NC_008312
Vibrio fisheri ES114	Vfish	NC_006840, NC_006841
Vibrio vulnificus YJ016	Vvuln	NC_005139, NC_005140
Xanthomonas campestris pv. vesicatoria str. 85-10	Xcamp	NC_007508
Xantomonas oryzae pv. Oryzae MAFF 311018	Xoryz	NC_007705
Yersinia pestis CO92	Ypes1	NC_003143
Yersinia pestis Nepal516	Ypes2	NC_008149

ARCHAEA (RefSeq 27.09.2006)	Abbreviation	Accession no
Aeropyrum pernix K1	Apern	NC_000854
Archaeoglobus fulgidus DSM 4304	Afulg	NC_000917
Haloarcula marismortui ATCC 43049	Hmari	NC_006396, NC_006397
Halobacterium sp. NRC-1	Halob	NC_002607
Haloquadratum walsbyi DSM 16790	Hwals	NC_008212
Methanocaldococcus jannaschii DSM 2661	Mjann	NC_000909
Methanococcoides burtonii DSM 6242	Mburt	NC_007955
Methanococcus maripaludis S2	Mmari	NC_005791
Methanopyrus kandleri AV19	Mkand	NC_003551
Methanosarcina acetivorans C2A	Macet	NC_003552
Methanosarcina barkeri str. fusaro	Mbark	NC_007355
Methanosarcina mazei Go1	Mmaze	NC_003901
Methanosphaera stadtmanae DSM 3091	Mstad	NC_007681
Methanospirillum hungatei JF-1	Mhung	NC_007796
Methanothermobacter thermautotrophicus str. Delta H	Mther	NC_000916
Nanoarchaeum equitans Kin4-M	Nequi	NC_005213
Natronomonas pharaonis DSM 2160	Nphar	NC_007426
Picrophilus torridus DSM 9790	Ptorr	NC_005877
Pyrobaculum aerophilum str IM2	Paero	NC_003364
Pyrococcus abyssi GE5	Pabys	NC_000868
Pyrococcus furiosus DSM 3638	Pfuri	NC_003413
Pyrococcus horikoshii OT3	Phori	NC_000961
Sulfolobus acidocaldarius DSM 639	Sacid2	NC_007181
Sulfolobus solfataricus P2	Ssolf	NC_002754
Sulfolobus tokodaii str. 7	Stoko	NC_003106
Thermococcus kodakarensis KOD1	Tkoda	NC_006624
Thermoplasma acidophilum DSM 1728	Tacid	NC_002578
Thermoplasma volcanium GSS1	Tvolc	NC_002689

EYKARYOTES (RefSeq 18.10.2006)	Abbreviation	Accession no
Candida glabrata	Cglab	NC_005967, NC_005968, NC_006026, NC_006027, NC_006028, NC_006029, NC_006030, NC_006031, NC_006032, NC_006033, NC_006034, NC_006035, NC_006036
Cryptococcus neoformans var. Neoformans JEC21	Cneof	NC_006670, NC_006679, NC_006680, NC_006681, NC_006682, NC_006683, NC_006684, NC_006685, NC_006686, NC_006687, NC_006691, NC_006692, NC_006693, NC_006694
Homo sapiens (ENSEMBL coding sequences 18.10.2006)	Hsapi	
Kluyveromyces lactis NRRL Y-1440	Klact	NC_006037, NC_006038, NC_006039, NC_006040, NC_006041, NC_006042
Saccharomyces cerevisiae S288c	Scere	NC_001133, NC_001134, NC_001135, NC_001136, NC_001137, NC_001138, NC_001139, NC_001140, NC_001141, NC_001142, NC_001143, NC_001144, NC_001145, NC_001146, NC_001147, NC_001148
Schizosaccharomyces pombe 972h-	Spomb	NC_003421, NC_003423, NC_003424
Yarrowia lipolytica	Ylipo	NC_006067, NC_006068, NC_006069, NC_006070, NC_006071, NC_006072
